# A Programmable Nanoreactor Orchestrates Cascade of DNA Sensing to Amplify cGAS‐STING Activation for Cancer Immunotherapy

**DOI:** 10.1002/advs.202518356

**Published:** 2026-01-20

**Authors:** Shuang Liang, Yiwei Tian, Feiyu Zhao, Yue Han, Kongshuo Ma, Linna Hai, Kaiqing Yun, Yueyang Zhao, Siqi Zhang, Ziyi Zhang, Yuxuan Peng, Kuan Hu, Jing Zhong, Bai Xiang, Zhaohui Wang

**Affiliations:** ^1^ State Key Laboratory of Bioactive Substance and Function of Natural Medicines Institute of Materia Medica Chinese Academy of Medical Sciences & Peking Union Medical College Beijing P. R. China; ^2^ Beijing Key Laboratory of Key Technologies for Natural Drug Delivery and Novel Formulations Institute of Materia Medica Chinese Academy of Medical Sciences & Peking Union Medical College Beijing P. R. China; ^3^ Hebei Key Laboratory of Innovative Drug Research and Evaluation School of Pharmaceutical Sciences Hebei Medical University Shijiazhuang P. R. China; ^4^ Department of Radiation Therapy Oncology Medical Department the Fifth Medical Center of Chinese People's Liberation Army General Hospital Beijing P. R. China; ^5^ School of Instrumentation and Opto‐Electronic Engineering Ministry of Education Key Laboratory of Precision Opto‐Mechatronics Technology Beihang University Beijing P. R. China

**Keywords:** cGAS‐STING pathway, immunotherapy, nanoparticles, telomere stress, TIM‐3 blockade

## Abstract

The cGAS‐STING pathway, a critical cytosolic DNA‐sensing mechanism in innate immunity, holds significant promise for cancer immunotherapy. However, conventional DNA‐damaging therapies lack tumor specificity and cause damage to normal tissue. Furthermore, dendritic cells (DCs), central to the STING‐mediated immune response, exhibit extrinsic immunosuppression via inhibitory receptors such as T‐cell immunoglobulin and mucin‐domain containing‐3 (TIM‐3), which impairs DNA internalization and subsequent pathway activation. Herein, we engineered a telomere stress‐induced nanoreactor composed of a pH‐responsive zeolitic imidazolate framework‐8 encapsulating telomerase‐targeted 6‐thio‐2’‐deoxyguanosine (6‐thio‐dG), with TIM‐3 antibodies (αTIM‐3) adsorbed onto its surface. Following accumulation in the tumor, the nanoreactor degrades within the acidic tumor microenvironment, releasing 6‐thio‐dG to induce tumor cell‐specific telomeric DNA damage. Concurrently, the αTIM‐3 blocks TIM‐3 receptors on DCs, thereby enhancing their internalization of the released DNA. This dual‐action strategy drives robust cGAS‐STING activation, enhancing type I interferon production and DCs maturation. In murine models of immunogenic and poorly immunogenic tumors, the nanoreactor significantly suppresses tumor growth and prolongs survival. By coupling tumor‐intrinsic telomere stress with DC‐extrinsic checkpoint inhibition, this work establishes a precision platform for cGAS‐STING pathway activation, presenting a promising therapeutic strategy for telomerase‐positive malignancies.

## Graphical abstract

A programmable nanoreactor was engineered for precise cGAS‐STING pathway activation in cancer immunotherapy. This strategy bridges targeted tumor DNA damage with DC immunomodulation, providing a translatable platform to unlock innate and adaptive antitumor immunity.

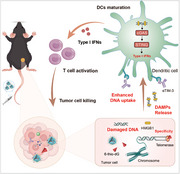

## 1 Introduction

Immunotherapy has revolutionized cancer treatment and improved outcomes for patients with various malignancies [1, 2]. Current immunotherapeutic strategies mainly focus on enhancing adaptive immunity through immune checkpoint inhibitors targeting inhibitory pathways or activating effector pathways through adoptive cell therapies [3, 4, 5]. Despite unprecedented clinical successes, these approaches benefit only a subset of patients, underscoring the critical need to identify alternative immunotherapeutic strategies.

Emerging evidence highlights the central role of innate immune pathways in bridging innate and adaptive immunity to enhance anti‐tumor responses [6, 7]. The cyclic GMP‐AMP synthase (cGAS)‐stimulator of interferon genes (STING) pathway, functioning as a cytosolic DNA sensor, is a pivotal component of the innate immune system. Once activated, it initiates a complex cascade of signaling events that culminates in the production of type I interferons (IFN‐I) and other pro‐inflammatory cytokines [8, 9, 10, 11, 12]. These immune mediators alert the immune system and ultimately orchestrate a robust adaptive immune response [13]. Therefore, the cGAS‐STING pathway represents a promising target for overcoming resistance to current immunotherapies.

Despite its promise, the activation of the cGAS‐STING pathway is fraught with multiple challenges. A primary limitation is the scarcity of immunogenic DNA required to trigger this pathway [14]. Recent studies highlight the importance of DNA‐damaging therapies, such as chemotherapy, radiotherapy, and reactive oxygen species‐generating treatments, to activate the cGAS‐STING pathway [15, 16, 17, 18, 19, 20]. However, their non‐selective nature often leads to toxicity in normal cells or even immune effector cells, which may paradoxically compromise antitumor immunity. Furthermore, dendritic cells (DCs), as the key antigen‐presenting cells responsible for cGAS‐STING‐driven immune activation, are critically regulated by immunosuppressive signals in the tumor microenvironment (TME) [21]. In particular, the inhibitory receptor T‐cell immunoglobulin and mucin‐domain containing‐3 (TIM‐3) has emerged as a major extrinsic immunosuppressive mechanism in DCs [22]. TIM‐3 expression on DCs impairs phagocytic function and restricts the internalization of tumor‐derived DNA, thereby blunting downstream cGAS‐STING pathway activation and limiting cross‐priming of T cells [23]. This receptor‐mediated dysfunction represents a key adaptive resistance mechanism that compromises the efficacy of STING‐targeted therapies. Given these challenges, there is an urgent need for innovative strategies that not only enable tumor‐specific DNA release but also promote efficient DC internalization and sensing by concurrently modulating extrinsic immunosuppressive pathways.

Telomerase, a reverse transcriptase that elongates telomeric DNA using an RNA template, is overexpressed in >85% of human tumors but is inactive in most normal cells. This distinct expression profile makes telomerase and telomeres attractive targets for precision cancer therapies [24, 25]. 6‐thio‐2'‐deoxyguanosine (6‐thio‐dG), a nucleoside analog structurally similar to deoxyguanosine, is preferentially incorporated into telomeric DNA during replication, causing chain termination and telomere dysfunction [26, 27]. Eventually, this process releases telomeric DNA fragments into the cytosol and induces tumor cell death via telomere damage, offering a precision therapeutic strategy for telomerase‐positive tumors. However, 6‐thio‐dG faces significant pharmacokinetic challenges such as short half‐life and poor tumor targeting efficiency [28]. These limitations underscore the need for nanocarriers to improve the therapeutic benefits.

Metal‐organic frameworks (MOFs), a class of coordination polymers constructed from metal ions or clusters and organic linkers, have garnered significant interest in drug delivery due to their high porosity and structural tunability [29]. Among the diverse MOF family, zeolitic imidazolate framework‐8 (ZIF‐8) stands out as a particularly promising nanoplatform, characterized by high biocompatibility and pH‐responsive degradation [30]. The mildly acidic nature of the TME can be leveraged to trigger controlled drug release from ZIF‐8. This property underscores the utility of ZIF‐8 as a versatile carrier for drug encapsulation and stimuli‐responsive delivery. Furthermore, ZIF‐8 can be synthesized via a straightforward one‐pot procedure in the presence of small‐molecule drugs [31]. The surface of ZIF‐8 also allows for functionalization with targeting ligands such as antibodies, proteins, or other macromolecules, thereby improving tumor‐specific delivery [32]. Collectively, these attributes affirm the excellent suitability of ZIF‐8 as a drug delivery vehicle.

**SCHEME 1:**
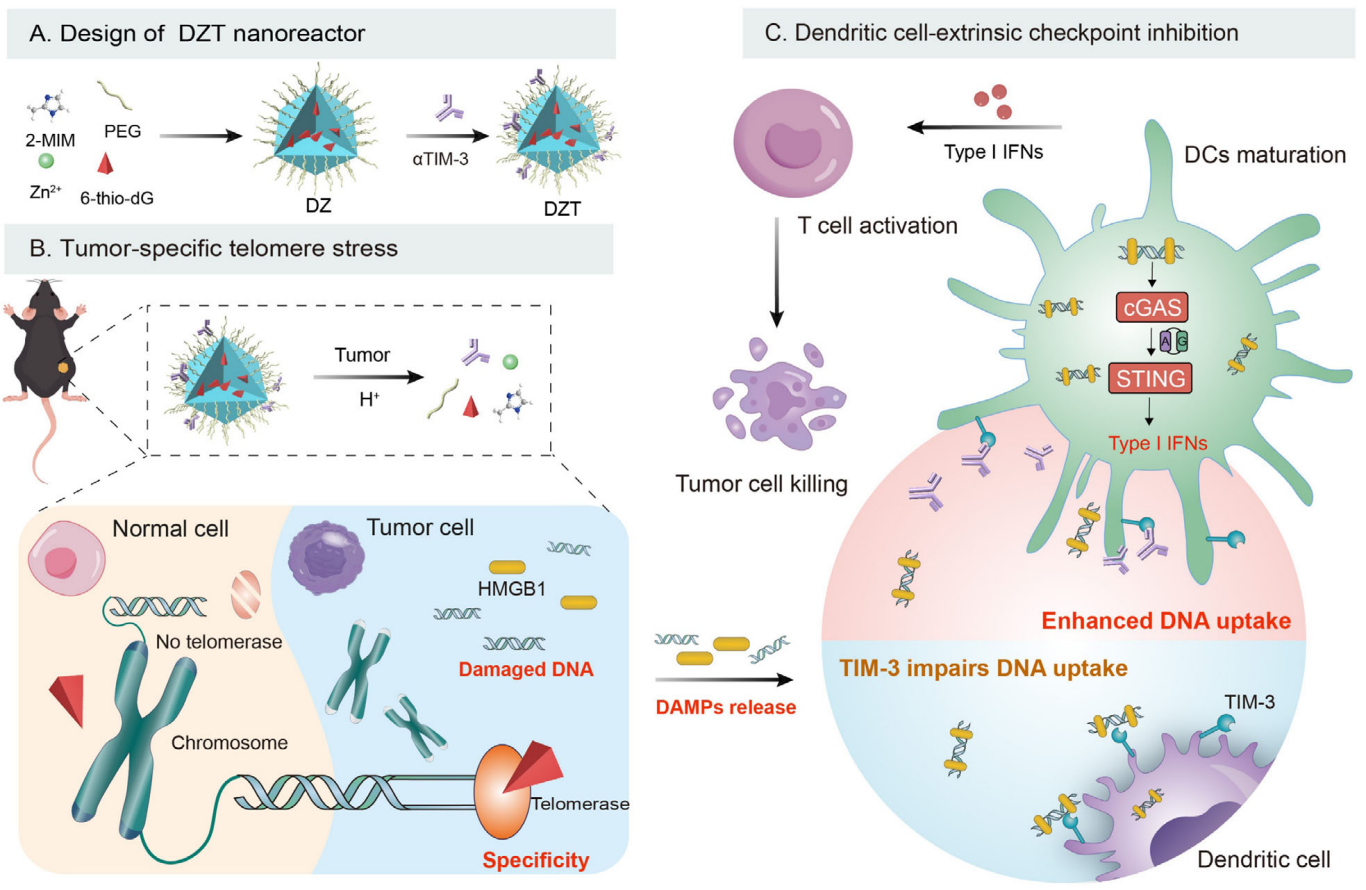
Schematic illustration of the fabrication of DZT and its mechanism for amplifying cGAS‐STING pathway activation.

In this study, we present a telomere stress‐induced nanoreactor using ZIF‐8 loaded with 6‐thio‐dG and further functionalized with TIM‐3 antibodies (αTIM‐3), which orchestrates a cascade of DNA sensing to amplify cGAS‐STING activation for enhancing anti‐tumor immune responses (Scheme 1). In detail, 6‐thio‐dG@ZIF‐8 (DZ) was obtained by a one‐pot method, followed by the loading of αTIM‐3 onto the surface of DZ to form the nanoreactor (DZT). When the nanoreactor reached the TME, ZIF‐8 enabled pH‐responsive release of the two cargoes. The released 6‐thio‐dG induces telomeric DNA damage of tumor cells, while αTIM‐3 overcomes uptake inhibition of DCs for efficient DNA sensing, amplifying STING pathway activation and downstream IFN‐I production. By spatially and temporally coupling tumor‐specific DNA release with DC reprogramming, the nanoreactor elicits robust immune responses and effectively inhibits tumor growth in both immunogenic and poorly immunogenic tumor‐bearing mice. This strategy unlocks the full potential of cGAS‐STING activation while minimizing systemic toxicity, positioning DZT as a versatile platform for numerous telomerase‐positive malignancies.

## 2 Results and Discussion

### 2.1 Preparation and Characterization of DZT Nanoreactor

**FIGURE 1:**
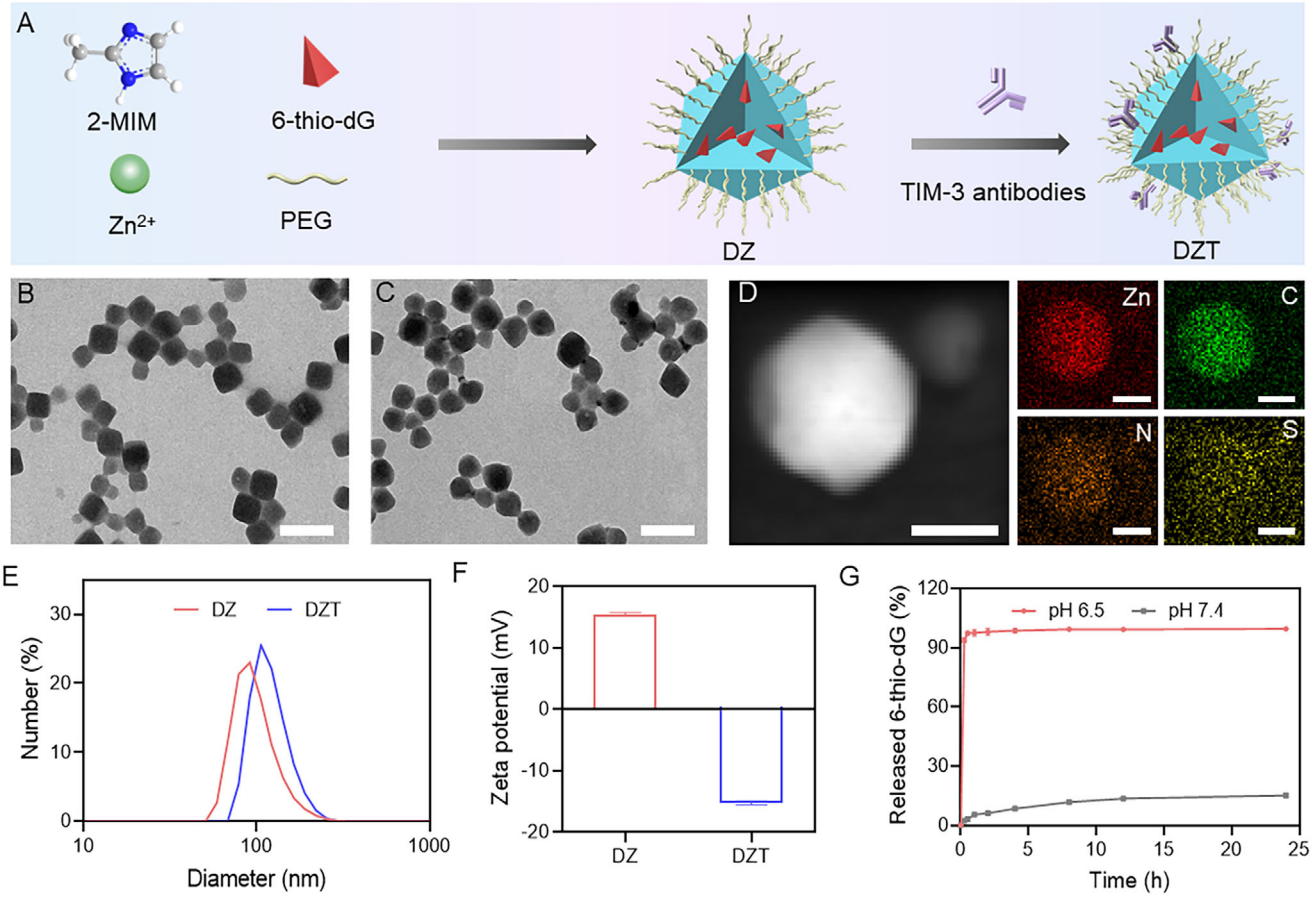
Synthesis and characterization of DZT nanoreactor. (A) Schematic diagram of the synthesis process of DZT. (B) TEM image of DZ NPs. Scale bar = 200 µm. (C) TEM image of DZT NPs. Scale bar = 200 µm. (D) Elemental mapping images of DZ NPs. Scale bar = 50 µm. The hydrodynamic diameters (E) and zeta potential (F) of ZIF‐8, DZ, and DZT. (G) The release profiles of 6‐thio‐dG in DZT cultured with different conditions. Data are shown as mean ± SD (n = 3). Statistical analysis was measured by one‐way ANOVA, ^*^ *p* < 0.05, ^**^ *p* < 0.01, and ^***^ *p* < 0.001.

DZ nanoparticles (NPs) were synthesized using a one‐pot method [33]. Briefly, 2‐methylimidazole was mixed with 8‐arm‐PEG and 6‐thio‐dG, followed by the addition of Zn(NO_3_)_2_ aqueous solution. The DZT nanoreactor was subsequently obtained through the electrostatic adsorption of αTIM‐3 onto the DZ NPs. Transmission electron microscopy (TEM) and scanning electron microscopy (SEM) images revealed that the DZ NPs exhibited a uniform morphology with an average diameter of approximately 100 nm (Figure 1B; Figure S1). Elemental mapping confirmed the homogeneous distribution of Zn, C, N, and S within the DZ NPs (Figure 1D), indicating the successful encapsulation of 6‐thio‐dG into the ZIF‐8 framework. After αTIM‐3 adsorption, no significant morphological changes were observed (Figure 1C), confirming the structural integrity of the nanoreactor. Dynamic light scattering (DLS) analysis indicated an increase in the average hydrodynamic diameter from 90 to 105 nm following αTIM‐3 functionalization (Figure 1E). Furthermore, the zeta potential shifted from +15.4 mV for DZ to −15.3 mV for DZT (Figure 1F), indicating the efficient adsorption of the negatively charged antibody. The loading content of 6‐thio‐dG and αTIM‐3 was determined to be 6.53% and 8.57%, as quantified by liquid‐phase analysis and gel electrophoresis, respectively (Figure S2). Powder X‐ray diffraction confirmed the preserved crystalline structure of ZIF‐8 after drug loading (Figure S3). DLS analysis revealed negligible size changes of DZT NPs in water, saline, and serum within 7 days (Figure S4), demonstrating excellent colloidal stability.

The in vitro release profiles of 6‐thio‐dG and αTIM‐3 were evaluated under both physiological and acidic conditions. At pH 6.5, approximately 100% of 6‐thio‐dG was released within 24 h, compared to only 16.8% at pH 7.4 (Figure 1G), indicating a pH‐dependent release profile. Similarly, the release of αTIM‐3 at pH 6.5 and 7.4 over 4 h was 92.6% and 14.5%, respectively (Figure S5), suggesting both 6‐thio‐dG and αTIM‐3 remained stably encapsulated during circulation and promoted release in acidic TME. Collectively, these results underscore the potential of the DZT nanoreactor as a potent nano‐immunomodulator for cancer immunotherapy.

### 2.2 Tumor‐Specific Telomere Stress and Immunogenic Cell Death

To evaluate the tumor‐specific cytotoxicity and the ability of DZ NPs to induce immunogenic cell death (ICD), we first investigated their cellular uptake in telomerase‐positive tumor cells. For this purpose, Cy5‐labeled DZ NPs were incubated with MC38 cells. Both fluorescence microscopy and flow cytometry revealed intense red fluorescence inside the cells after 12 h of incubation (Figure S6), indicating efficient and time‐dependent internalization of the NPs. To elucidate the internalization pathway, MC38 cells were pretreated with specific inhibitors targeting distinct endocytic mechanisms before adding the DZ NPs. As shown in Figure S7, cellular uptake was significantly reduced to 54.9% and 65.8% of the control level in the presence of amiloride (AMR) and methyl‐β‐cyclodextrin (mβ‐CD), respectively. This suggests that the NPs are primarily internalized via macropinocytosis and caveolae‐mediated endocytosis [34, 35].

**FIGURE 2:**
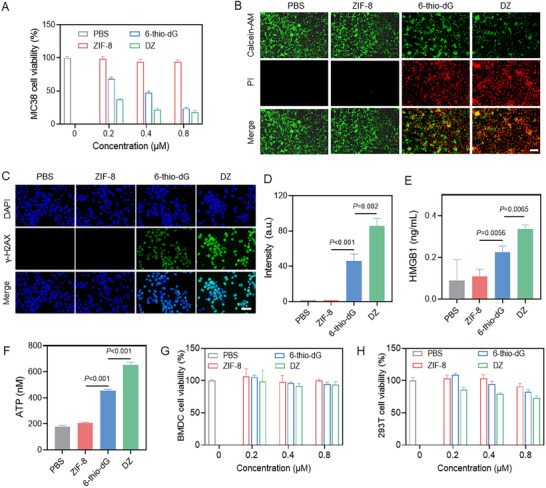
Tumor‐specific telomere stress and ICD induced by DZ. (A) The cytotoxicity of PBS, ZIF‐8, 6‐thio‐dG, and DZ NPs against MC38 cells. (B) Calcein AM/PI staining of MC38 cells treated with PBS, ZIF‐8, 6‐thio‐dG, and DZ NPs. Scale bar = 100 µm. (C) Fluorescence images of MC38 cells treated with PBS, ZIF‐8, 6‐thio‐dG, and DZ NPs for detecting intracellular DNA levels by γ‐H2AX probes. Scale bar = 50 µm. (D) Relative mean fluorescence intensity of the MC38 tumor stained with γ‐H2AX. (E) Detection of cytoplasmic HMGB1 by ELISA assay. (F) Detection of extracellular ATP by Luciferin‐based ATP assay kit. The cytotoxicity of PBS, ZIF‐8, 6‐thio‐dG, and DZ NPs against BMDC (H) and 293T cells (G). Data are shown as mean ± SD (n = 3). Statistical analysis was measured by one‐way ANOVA, ^*^ *p* < 0.05, ^**^ *p* < 0.01, and ^***^ *p* < 0.001.

We next evaluated cytotoxicity using 3‐(4,5‐Dimethylthiazol‐2‐yl)‐2,5‐diphenyltetrazolium bromide (MTT) assay. Free 6‐thio‐dG exhibited potent cytotoxicity against MC38 cells. Notably, while the ZIF‐8 carrier showed negligible cytotoxicity, the DZ NPs demonstrated significantly enhanced inhibitory effects compared to an equivalent concentration of free 6‐thio‐dG (Figure 2A). This underscores the critical role of the ZIF‐8 carrier in enhancing the delivery and potency of 6‐thio‐dG. This enhanced cytotoxicity was corroborated by live/dead cell staining assays (Figure 2B), which were in line with the MTT results. Furthermore, flow cytometry analysis with Annexin V‐FITC/propidium iodide (PI) co‐staining confirmed the induction of apoptosis by DZ NPs (Figure S8).

To investigate the potential of DZ NPs to induce ICD, we monitored the release of key damage‐associated molecular patterns (DAMPs), including high‐mobility group box 1 protein (HMGB1), adenosine triphosphate (ATP), and double‐strand DNA (dsDNA). To confirm that 6‐thio‐dG induces DNA damage, we detected levels of phosphorylated histone H2AX (γ‐H2AX), a marker of DNA double‐strand breaks. Fluorescence imaging revealed a stronger γ‐H2AX signal in DZ NP‐treated cells compared to the PBS‐treated group (Figure 2C,D), indicating extensive DNA double‐strand breaks. Enzyme‐linked immunosorbent assay (ELISA) further demonstrated a substantial increase in extracellular HMGB1 and ATP levels in the supernatant of MC38 cells treated with DZ NPs (Figure 2E,F), confirming robust ICD activation. To verify the tumor cell specificity of DZ NPs, we performed MTT assays on immune cells and non‐tumorigenic human epithelial cells. The results indicated that DZ NPs were minimally cytotoxic to these normal cell types (Figure 2G,H). These results collectively demonstrate that DZ NPs can not only elicit tumor‐specific cytotoxicity but also amplify immunogenic signaling through the release of DAMPs.

### 2.3 Enhanced cGAS‐STING Activation and DC Maturation

**FIGURE 3:**
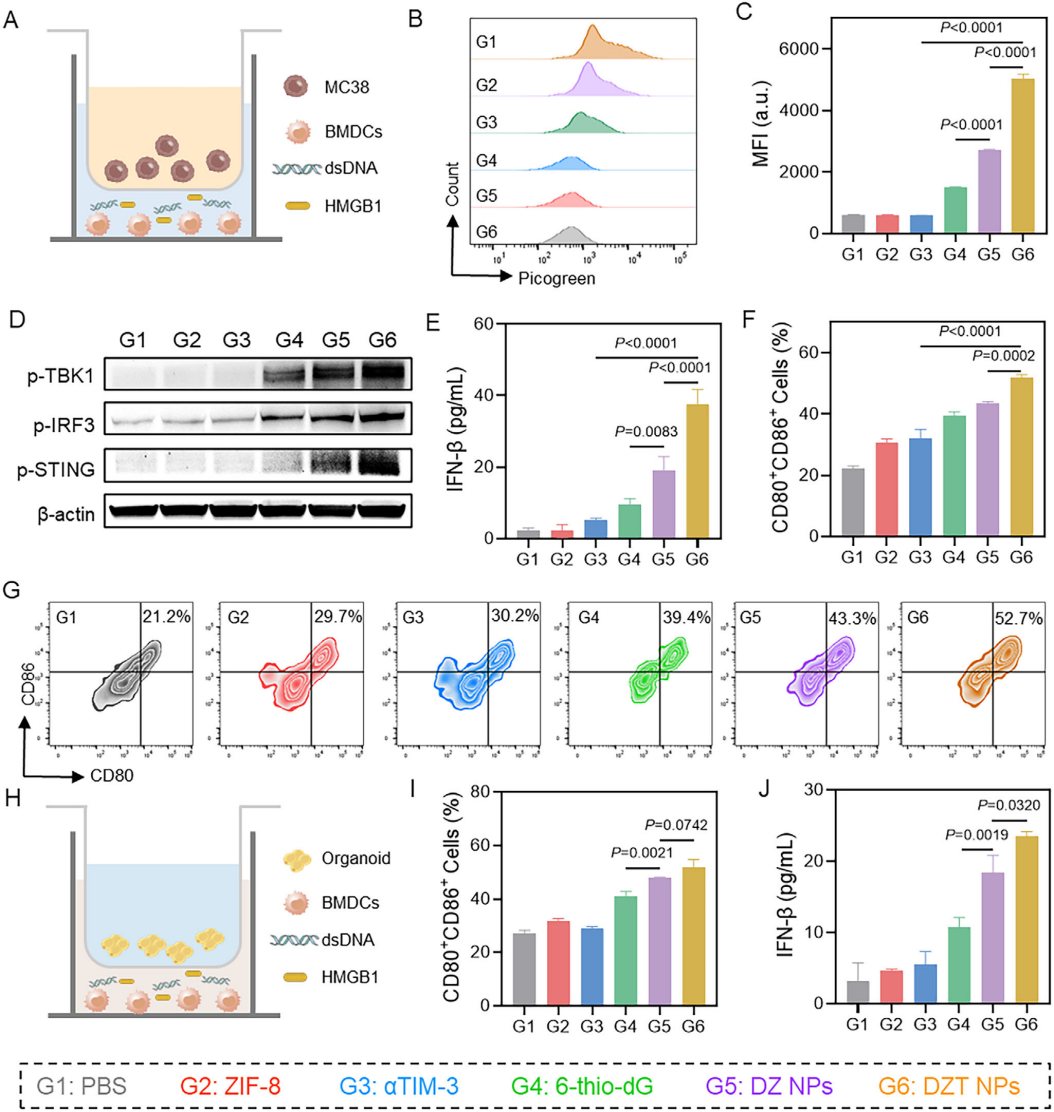
Detection of immune activation induced by DZT nanoreactor. (A) Schematic diagram of the transwell experiment to study tumor‐specific telomere stress for inducing cGAS‐STING signaling pathway activation and DC maturation. (B) Flow cytometry analysis of BMDCs stained with PicoGreen after co‐incubation with MC38 cells treated with different groups. (C) Relative mean fluorescence intensity of BMDCs stained with PicoGreen after co‐incubation with MC38 cells treated with PBS, ZIF‐8, 6‐thio‐dG, DZ NPs, and DZT NPs. (D) Western blot image of p‐STING, p‐IRF3, and p‐TBK1 in BMDCs after co‐incubation with MC38 cells treated with various treatments. (E) IFN‐β secretion levels in the supernatants of BMDCs after co‐incubation with MC38 cells treated with various treatments. The quantification (F) and flow cytometry analysis (G) of BMDC maturation after co‐incubation with MC38 cells treated with various treatments. (H) Schematic diagram of the transwell experiment for studying the activation of the cGAS‐STING signaling pathway and the maturation of DCs induced by breast cancer organoids. (I) The quantification of BMDC maturation after co‐incubation with organoids treated with various groups. (J) IFN‐β secretion levels in the supernatants of BMDCs after co‐incubation with organoids treated with various groups. Data are shown as mean ± SD (n = 3). Statistical analysis was measured by one‐way ANOVA, ^*^ *p* < 0.05, ^**^ *p* < 0.01, and ^***^ *p* < 0.001.

To investigate whether telomere stress‐induced DNA release activates the cGAS‐STING pathway, we first assessed DNA uptake in bone marrow‐derived dendritic cells (BMDCs) co‐cultured with MC38 cells using Transwell chambers [36]. The results indicated a moderate level of DNA uptake in the DZ group. Notably, the DZT group exhibited the highest level of DNA uptake (Figure 3B,C), which was attributed to the αTIM‐3 relieving TIM‐3‐mediated inhibition of phagocytosis. We next performed Western blot analysis to evaluate the activation of key proteins in the STING pathway [37, 38]. As shown in Figure 3D, ZIF‐8 exhibited minimal impact on the phosphorylation levels of STING, TBK1, and IRF3 (denoted as p‐STING, p‐TBK1, and p‐IRF3, respectively). In contrast, treatment with DZ NPs significantly increased the levels of p‐STING, p‐TBK1, and p‐IRF3. This activation was further enhanced by treatment with DZT NPs. Given that p‐IRF3 is a critical transcriptional regulator for IFN‐I production, we quantified IFN‐β secretion in the culture supernatants of BMDCs using ELISA (Figure 3E). The DZT NPs treatment group demonstrated the highest IFN‐β levels among all experimental groups. These results provide compelling evidence that telomere stress‐mediated DNA liberation effectively activates the cGAS‐STING signaling pathway, with optimal pathway stimulation achieved through the combinatorial therapeutic approach incorporating both 6‐thio‐dG and αTIM‐3 into ZIF‐8.

The maturation of DCs plays a pivotal role in facilitating antigen presentation and initiating adaptive immune responses. Quantitative analysis revealed that DZ NPs markedly enhanced the surface expression of co‐stimulatory molecules on BMDCs, with a dual‐positive CD80^+^CD86^+^ population frequency of 43.4% compared to 22.1% in the PBS group (Figure 3F,G). This pronounced upregulation of maturation markers demonstrates that 6‐thio‐dG‐mediated telomere stress serves as a potent trigger for DC activation. Furthermore, the immunostimulatory effect was amplified in the DZT group, suggesting synergistic contributions from αTIM‐3 blockade in optimizing DC functionality.

To further validate these results, we employed tumor organoids as a physiologically relevant model. Pretreated organoids were co‐cultured with BMDCs to assess STING pathway activation and DC maturation (Figure 3H–J; Figure S9). Consistent with the cell‐based assays, DZT NPs demonstrated superior efficacy in promoting both cGAS‐STING activation and DC maturation compared to all other treatment groups, highlighting its strong therapeutic potential. Collectively, these results underscore the capacity of DZT NPs to reprogram DCs and enhance their immunogenicity through a combination of telomere stress‐induced DNA release and the blockade of the TIM‐3 inhibitory pathway.

### 2.4 Mechanistic Analysis

**FIGURE 4:**
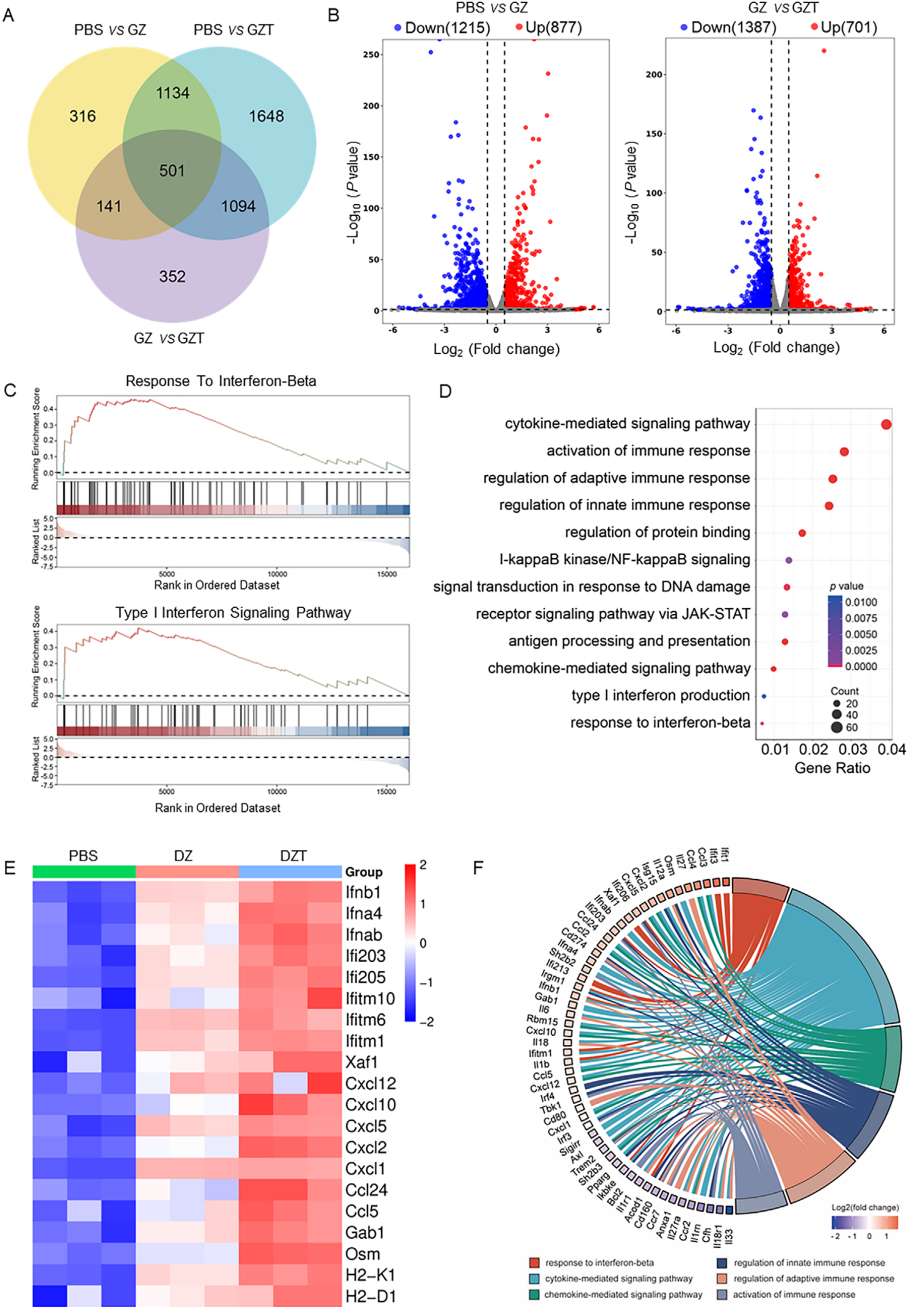
RNA‐sequencing analysis of immune activation induced by DZT nanoreactor. (A) Venn diagram showing the differential expression of genes (DEGs) between PBS, DZ, and DZT groups. (B) Volcano plot depicting the total DEGs between PBS, DZ, and DZT groups. (C) GSEA of DEGs between DZ and DZT groups. (D) KEGG pathway enrichment analysis of DEGs between DZ and DZT groups, with each bubble representing an enriched pathway. (E) Heatmap showing the expression of differentially expressed immune‐related genes in PBS, DZ, and DZT groups. (F) Chord diagram enriched immune response associated biological processes. The different colors represent the different categories to which they belong.

To elucidate the biological mechanisms underlying the synergistic effect of DZT, we performed comprehensive genome‐wide RNA sequencing on BMDCs subjected to various treatments. Venn diagram analysis identified differentially expressed genes (DEGs) among the PBS, DZ, and DZT treatment groups (Figure 4A). A core set of 501 genes exhibited overlapping expression profiles across groups, suggesting involvement in common regulatory pathways or biological functions. Figure 4B quantifies the distribution of up‐ and down‐regulated genes for each treatment group. Gene Set Enrichment Analysis (GSEA) revealed that immune‐related pathways, including the response to IFN‐β, were significantly enriched in the DZT group compared to the DZ group (Figure 4C). To further investigate how DZT enhances STING activation, Gene Ontology (GO) enrichment analysis was conducted on the DEGs. As shown in Figure 4D, DZT treatment markedly altered the expression of genes implicated in diverse biological processes, including immune responses, biological regulation, and cellular processes. Furthermore, Kyoto Encyclopedia of Genes and Genomes (KEGG) analysis demonstrated significant enrichment in the DZT group for pathways associated with DNA damage response and immune activation. These included cytokine‐mediated signaling, response to IFN‐β, chemokine‐mediated signaling, and cellular response to IFN‐β pathways, which are critically linked to STING pathway activation and broader immune responses. A differential gene clustering heatmap comparing PBS, DZ, and DZT groups (Figure 4E) highlights distinct transcriptional signatures induced by each treatment, further supporting the enhanced capacity of DZT to trigger cGAS‐STING pathway activation. A chord diagram illustrated the network of interactions between the key proteins and pathways identified in our analysis, providing a systems‐level view of the immune activation program induced by DZT (Figure 4F). Collectively, these results demonstrate that DZT effectively activates the cGAS‐STING pathway for tumor immunotherapy.

### 2.5 In Vivo Biosafety and Tumor Targeting

Having confirmed the effective in vitro cGAS‐STING pathway activation of DZT NPs, we next evaluated their in vivo safety profile. Specifically, healthy BALB/c mice were intravenously injected with DZT NPs at a dose of 10 mg/kg and monitored over a 14‐day period. Comprehensive toxicological assessments, including blood parameter analysis, body weight monitoring, and hematoxylin and eosin (H&E) staining of major organs, were performed. The blood biochemistry and whole blood analysis revealed no statistically significant alterations in key parameters (Figure S10). Consistent with hematological results, no significant changes in body weight were observed in treated mice compared to the control group (Figure S11). Histopathological examination of the heart, liver, spleen, lungs, and kidneys revealed no evidence of inflammation, necrosis, or structural lesions (Figure S12). Hemolysis assays demonstrated a hemolytic rate of less than 1% (Figure S13). In addition, we evaluated potential systemic inflammatory responses in mice by measuring key inflammatory cytokines, including IFN‐γ, IL‐6, and TNF‐α at 1 h post‐injection of DZ or DZT. The results indicated no significant acute inflammatory activation at this early time point in either group, suggesting a favorable biosafety profile with respect to avoidance of systemic inflammatory reactions (Figure S14). These results collectively support the compatibility of DZT for intravenous administration.

Subsequent biodistribution analysis using fluorescence imaging revealed preferential accumulation of DZT NPs at the tumor site, reaching a maximum 8 h post‐injection (Figure S15). Furthermore, *ex vivo* fluorescence imaging of the tumor and major organs confirmed the tumor targeting ability. Taken together, these results demonstrate that DZT NPs effectively target tumor tissue with remarkable in vivo biosafety.

### 2.6 Antitumor Efficacy in Immunogenic Cancer Model

**FIGURE 5:**
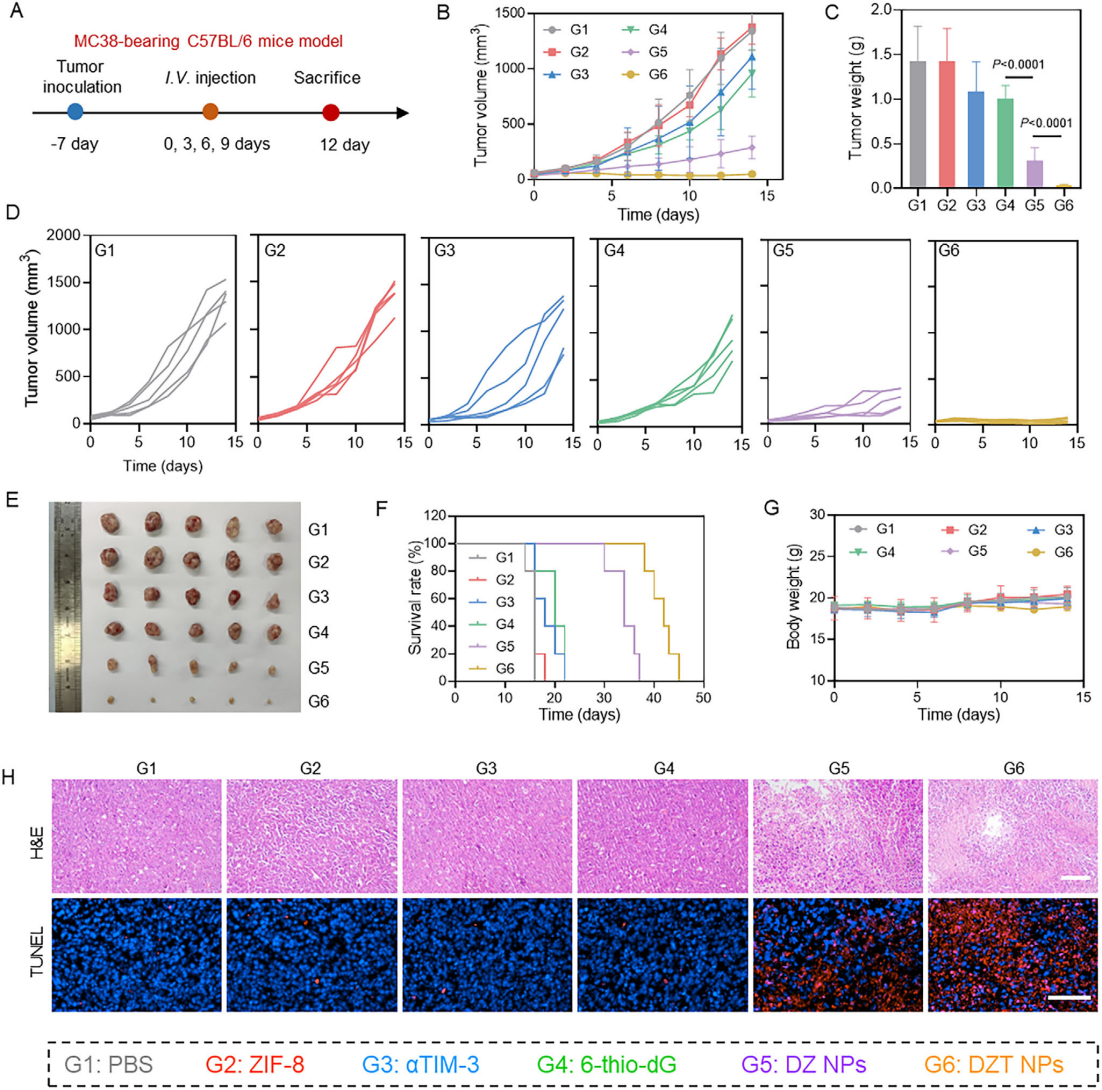
Antitumor effect of DZT NPs in the MC38‐bearing tumor model. (A) Scheme illustrating the establishment and treatment of the MC38‐bearing C57BL/6 mice model. (B) Tumor volume curves of MC38‐bearing C57BL/6 mice in different groups. (C) Tumor weights from mice with various treatments. (D) Individual tumor growth curves of MC38 tumor‐bearing mice with different treatments. (E) Representative digital images of the tumor after different treatments. (F) Survival curves of mice after different treatments. (G) Body weight of mice within the treatment period. (H) H&E and TUNEL staining in the MC38 tumor after different treatments. Scale bar = 100 µm. Data are shown as mean ± SD (n = 3). Statistical analysis was measured by one‐way ANOVA, ^*^ *p* < 0.05, ^**^ *p* < 0.01, and ^***^ *p* < 0.001.

Based on the promising in vivo safety profile and tumor‐targeting capability, we next evaluated the in vivo therapeutic efficacy of DZT NPs in the MC38 colon cancer model. As illustrated in Figure 5A, MC38 tumor cells were subcutaneously inoculated into the right flank of C57BL/6 mice. Once tumor volumes reached approximately 50 mm^3^, the mice were randomly assigned to six treatment groups: PBS, ZIF‐8, αTIM‐3, free 6‐thio‐dG, DZ NPs, or DZT NPs. The formulations were administered intravenously every three days for a total of four injections. The body weights and tumor volumes were monitored throughout the treatment period. Tumor growth curves (Figure 5B–D) showed that PBS‐treated mice exhibited rapid and progressive tumor growth. Treatment with free 6‐thio‐dG alone resulted in modest inhibitory effects compared to the PBS group, whereas DZ NPs demonstrated enhanced tumor suppression, underscoring the therapeutic advantage of drug delivery. Most notably, the DZT NPs group exhibited the most potent tumor growth inhibition, attributable to the critical role of αTIM‐3 blockade in enhancing DC uptake of tumor‐derived DNA and amplifying the subsequent cGAS‐STING pathway activation. These results were corroborated by tumor weight and representative photographs of excised tumors (Figure 5C,E).

Furthermore, mice in the DZT NP treatment group exhibited a significant survival benefit compared to all other groups (Figure 5F). H&E and terminal deoxynucleotidyl transferase dUTP nick end labeling (TUNEL) staining further confirmed the potent antitumor effects of DZT NPs (Figure 5H). Importantly, no significant body weight fluctuations or obvious histopathological damage of main organs were observed across all treatment groups (Figure 5G; Figure S16), confirming the excellent safety of the formulations. Collectively, these results establish the synergistic therapeutic benefits of integrating 6‐thio‐dG and αTIM‐3 into a nanoplatform.

### 2.7 Systemic Immune Response Evaluation

**FIGURE 6:**
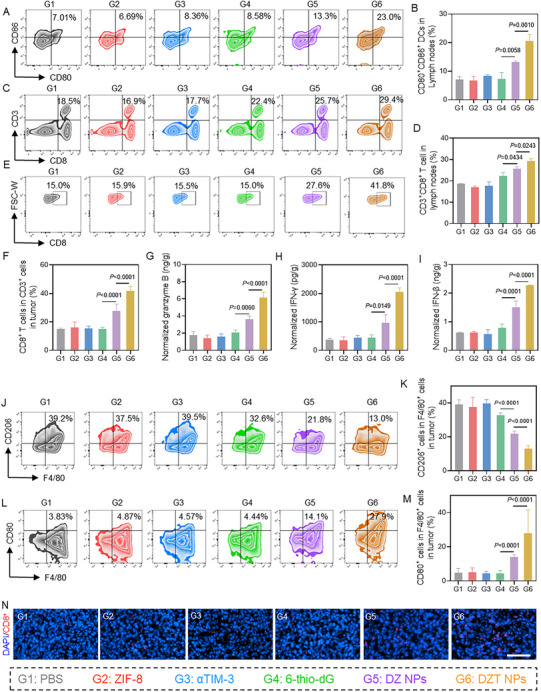
Composition of immune cells in the dLNs, spleens, and tumors after the mice were treated by DZT. Flow cytometric assay (A) and quantification (B) of mature DCs in the dLNs after different treatments. Representative flow cytometric analysis (C) and quantification (D) of CD8^+^ T cells in the dLNs after different treatments. Representative flow cytometric analysis (E) and quantification (F) of CD8^+^ T cells in tumor after different treatments. (G‐I) Serum cytokine concentrations of IFN‐γ, Granzyme B, and IFN‐β. Flow cytometry analysis (J) and quantification (K) of M2 (CD206^+^) macrophages. Flow cytometry analysis (L) and quantification (M) of M1 (CD80^+^) macrophages. (N) Immunofluorescence images of CD8^+^ T cell infiltration in tumor slices after different treatments. Scale bar = 100 µm. Data are shown as mean ± SD (n = 3). Statistical analysis was measured by one‐way ANOVA, ^*^ *p* < 0.05, ^**^ *p* < 0.01, and ^***^ *p* < 0.001.

To elucidate the antitumor mechanism of DZT NPs, we performed comprehensive immune profiling of tumor‐infiltrating immune cells from the draining lymph nodes (dLNs), spleens, and tumors of the treated mice. Given the pivotal role of DC maturation in initiating adaptive immunity, we first quantified CD11c^+^, CD80^+^, and CD86^+^ DCs within the dLNs. As shown in Figure 6A,B, the frequency of CD11c^+^CD80^+^CD86^+^ DCs in the DZT NP group reached 20.4%, representing a 1.6‐fold increase over the DZ NP group. This indicates that αTIM‐3 incorporation potently enhances DC maturation. We next focused on T cells given their critical role in antitumor immunity. As shown in Figure 6C,D, and Figure S17, the proportions of CD4^+^ and CD8^+^ T cells in the DZT group reached 42.3% and 29.7%, representing increases of 1.9‐fold and 4.4‐fold over the control group, respectively. Similarly, the percentage of CD4^+^ T and CD8^+^ T cells in the spleen increased markedly in the DZT NPs group (Figure S18). We also analyzed tumor‐infiltrating T cells. Mice treated with DZT NPs showed a substantial increase in the number of both CD4^+^ and CD8^+^ T cells within the tumor tissue (Figure 6E,F; Figure S19). The enhanced intratumoral infiltration of CD8^+^ T cells was also demonstrated by immunofluorescence assay (Figure 6N). Furthermore, treatment with DZT NPs markedly increased the secretion of the cytotoxic mediators interferon‐γ (IFN‐γ) and granzyme B (Figure 6G,H). These results indicate that DZT NPs can effectively activate T cells and thereby achieve tumor‐killing effects.

Systemic STING pathway activation was assessed by measuring serum cytokine levels using ELISA. As shown in Figure 6I, free 6‐thio‐dG induced only a modest increase in serum IFN‐β, which was not significantly different from the PBS group. In contrast, mice treated with DZT NPs exhibited a significant increase in serum IFN‐β compared to all other groups, indicating robust activation of the cGAS‐STING pathway. Moreover, the variation in the proportion of M2 polarization (CD206^+^) presents an opposite trend, evidenced by a sharp decline from 38.6% to 12.7% (Figure 6J,K). Concurrently, the proportion of CD80^+^ macrophages was significantly upregulated (Figure 6L,M), reflecting a successful reprogramming of the TME toward an immunostimulatory state in DZT NP‐treated mice. These results suggest that DZT NPs orchestrate a potent antitumor immune response by enhancing DC maturation and triggering robust T cell activation to achieve effective tumor growth inhibition and prolonged survival in tumor‐bearing mice.

### 2.8 Antitumor Efficacy and Immune Response in Poorly Immunogenic Cancer Model

**FIGURE 7:**
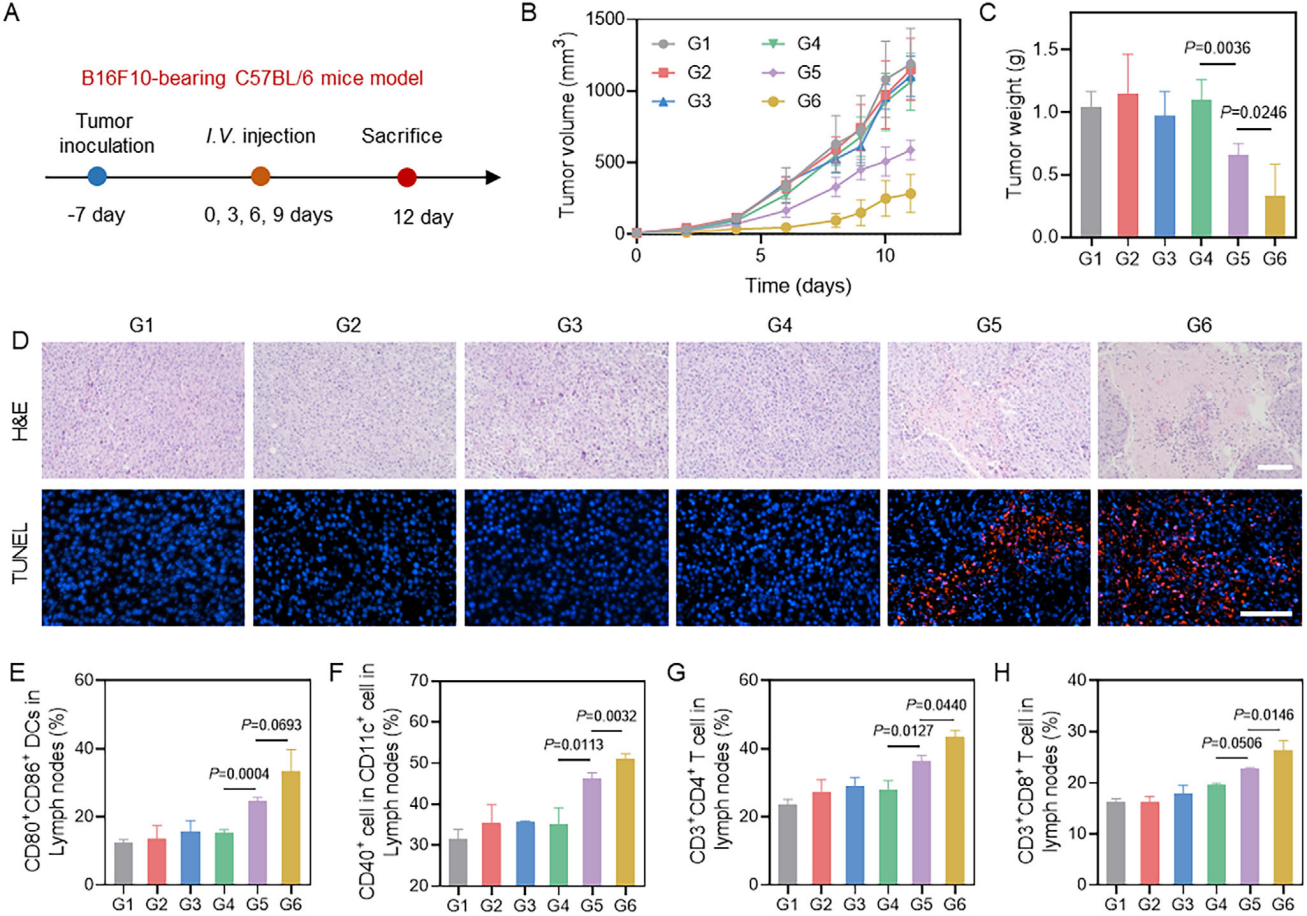
Antitumor effect of DZT NPs in B16F10‐bearing tumor model. (A) Scheme illustrating the establishment and treatment of the B16F10‐bearing C57BL/6 mice model. (B) Tumor volume curves of B16F10‐bearing C57BL/6 mice in different groups. (C) Tumor weights from mice with various treatments. (D) H&E and TUNEL staining in the MC38 tumor after different treatments. Scale bar = 100 µm. (E, F) The quantification of mature DCs in the dLNs after different treatments. (G, H) The quantification of CD8^+^ and CD4^+^ T cells in the dLNs after different treatments. Data are shown as mean ± SD (n = 3). Statistical analysis was measured by one‐way ANOVA, ^*^ *p* < 0.05, ^**^ *p* < 0.01, and ^***^ *p* < 0.001.

To evaluate the broad applicability of the therapeutic platform, we evaluated the efficacy of DZT NPs in an aggressive and poorly immunogenic B16‐F10 melanoma model. Whereas free 6‐thio‐dG showed negligible therapeutic effect, DZT NPs achieved significant tumor growth suppression (Figure 7B–D; Figures S20 and S21), demonstrating their therapeutic advantage even in this challenging setting. Importantly, all treatment groups maintained stable body weights with no significant histopathological changes in major organs (Figures S22 and S23), confirming the excellent biosafety profile of DZT NPs.

**FIGURE 8:**
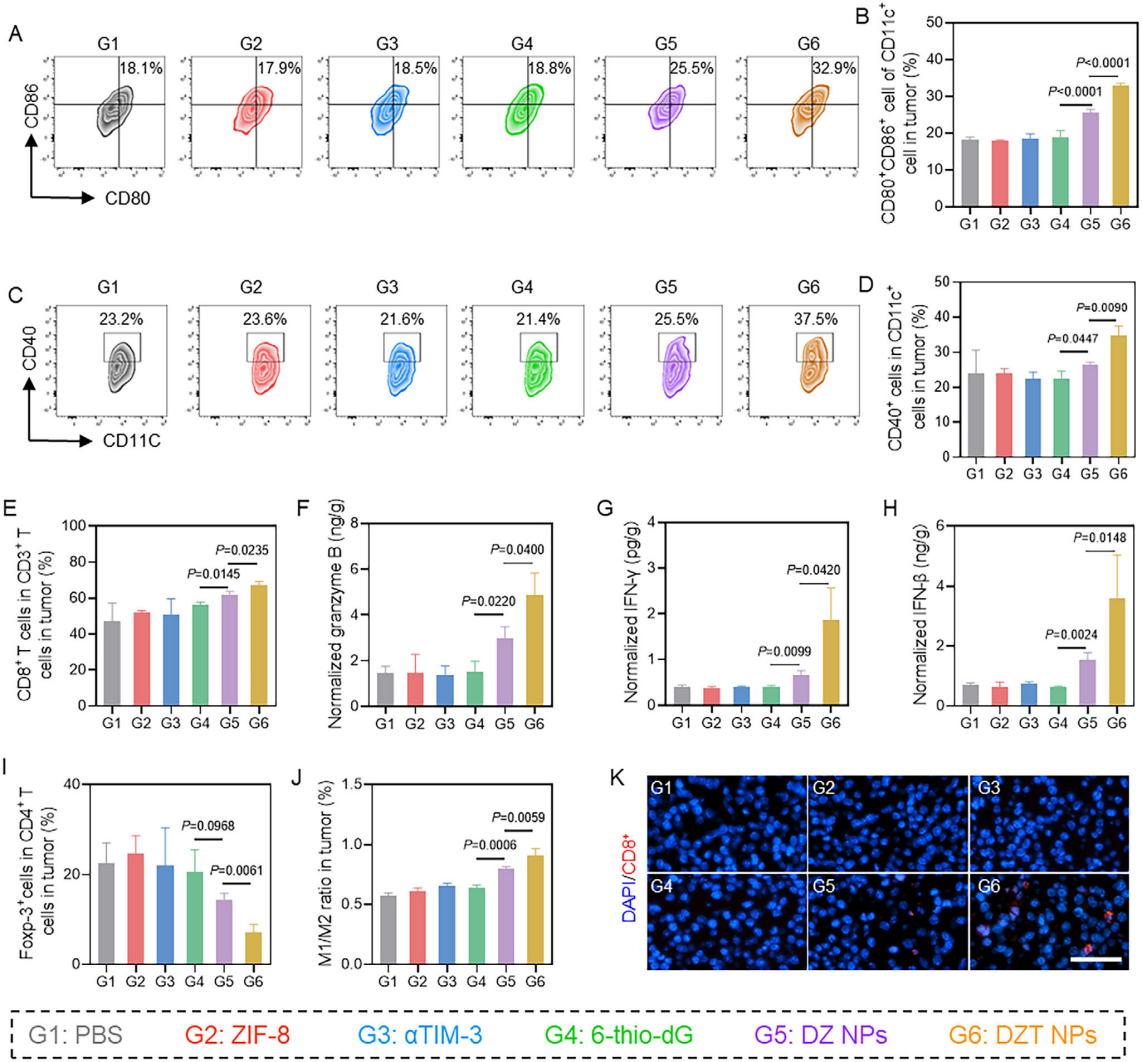
Immune responses against tumor therapies. Representative flow cytometric analysis (A) and quantification (B) of CD80^+^CD86^+^ expression in TME after different treatments. Representative flow cytometric analysis (C) and quantification (D) of CD40^+^ expression in TME. (E) The quantification of CD8^+^ T cells in the tumor after different treatments. (F–H) Serum cytokine concentrations of Granzyme B, IFN‐γ, and IFN‐β. (I) The percentages of FoxP3^+^ T cells (Tregs) among CD4^+^ T cells infiltrated within TME. (J) The M1/M2 ratio in TME. (K) Immunofluorescence images of CD8^+^ T cell infiltration in tumor slices after different treatments. Scale bar = 100 µm. Data are shown as mean ± SD (n = 3). Statistical analysis was measured by one‐way ANOVA, ^*^ *p* < 0.05, ^**^ *p* < 0.01, and ^***^ *p* < 0.001.

Mechanistically, DZT induced the maturation of DCs in dLNs, demonstrated by enhanced expression of CD86^+^CD80^+^ and CD40^+^ costimulatory molecules (Figure 7E,F; Figures S24 and S25). In addition, both CD4^+^ and CD8^+^ T cells in the lymph nodes and spleen were remarkably increased (Figure 7G,H; Figures S26–S29), contributing to enhanced immune‐mediated cytotoxicity. We also evaluated tumor‐infiltrating DCs, which are crucial for sustaining T‐cell activity within the TME (Figure 8A–D). Consistent with the dLN data, free 6‐thio‐dG did not induce appreciable changes, whereas DZT NP treatment significantly increased the expression of DC maturation markers. Meanwhile, DZT NPs effectively increased the percentage of CD8^+^ T cells among tumor‐infiltrating T cells compared to control groups (Figure 8E,K; Figure S30), and subsequently induced their priming and proliferation to enter the effector period characterized by the secretion of granzyme B and IFN‐γ (Figure 8F,G). The secretion of IFN‐β in different treatment groups reflects the activation of the cGAS‐STING pathway (Figure 8H). Furthermore, DZT NPs reduced the frequency of regulatory T cells (Tregs) (Figure 8I; Figure S31) and promoted the repolarization of M2‐like tumor‐associated macrophages toward an M1‐like phenotype (Figure 8J). These changes demonstrate that the nanoreactor alleviates immunosuppression within the TME, thereby enhancing antitumor immunity.

These results collectively establish DZT NPs as a potent immunomodulatory nanoplatform capable of overcoming the immunosuppression of poorly immunogenic tumors, demonstrating consistent efficacy across diverse TMEs.

## 3 Conclusion

In summary, we have developed a telomere stress‐induced nanoreactor that integrates tumor‐specific DNA damage with DC immunomodulation to potentiate cGAS‐STING pathway activation. The nanoreactor leverages a pH‐responsive ZIF‐8 to co‐deliver 6‐thio‐dG and αTIM‐3 into the TME. Upon acidic TME‐triggered degradation, 6‐thio‐dG selectively incorporates into telomeric DNA of tumor cells, inducing telomere dysfunction and cytosolic release of immunogenic DNA fragments. Concurrently, the αTIM‐3 counteracts DC‐extrinsic immunosuppression by blocking the TIM‐3 receptor, thereby enhancing the internalization of the released DNA and amplifying the cGAS‐STING pathway activation. In vivo evaluations in both immunogenic and poorly immunogenic models demonstrated potent tumor suppression. This work advances the frontier of cancer immunotherapy by bridging targeted DNA damage with immune reprogramming, offering a versatile platform to unlock the full potential of innate and adaptive antitumor immunity.

## 4 Experimental Section

### 4.1 Materials

8‐arm PEG‐OH‐40K was purchased from Sinopeg. 2‐Methylimidazole, Zn(NO_3_)_2_·6H_2_O, NaOH and Tris(hydroxymethyl)aminomethane were purchased from Aladdin. 6‐thio‐dG was purchased from Bide Pharmatech. Anti‐mouse TIM‐3(CD366) was purchased from Bio X Cell (USA). Cy5‐NHS ester was purchased from OKeanos. PBS, DMEM (high glucose), and RPMI 1640 were purchased from Zhongke Maichen. Fetal bovine serum (FBS) was purchased from Sigma. Penicillin/streptomycin was purchased from Pricella. Trypsin‐EDTA Solution, Red Blood Cell Lysis Buffer, TBST buffer, and Mouse HMGB1 ELISA Kit were purchased from Solarbio. GM‐CSF and IL‐4 were purchased from Pepro Tech. Hochest 33342, CCK‐8, Calcein AM/PI Cell Viability/Cytotoxicity Assay Kit, Annexin V‐FITC/PI Apoptosis Detection Kit, ATP Assay Kit, DNA Damage Assay Kit by γ‐H2AX Immunofluorescence, and BCA protein assay kit were purchased from Beyotime. RIPA lysis buffer and β‐actin Mouse mAb were purchased from ImmunoWay. SDS‐PAGE Sample Loading Buffer was purchased from Genscript. Phospho‐NF‐κB p65 Rabbit mAb, Phospho‐STING Rabbit mAb, Phospho‐TBK1 Rabbit mAb, and Phospho‐IRF‐3 Rabbit mAb were purchased from Cell Signaling Technology. HRP‐conjugated Goat Anti‐Rabbit IgG was purchased from APExBIO. HRP‐conjugated Rabbit Anti‐mouse IgG was purchased from BBI. Mouse IFN‐β ELISA Kit was purchased from Elabscience. Mouse IL‐6 Uncoated ELISA Kit, Mouse IFN‐γ Uncoated ELISA Kit, Mouse TNF‐α Uncoated ELISA Kit, anti‐CD45‐Alex Fluor 700, anti‐CD11c‐PerCP‐Cy5.5, anti‐CD80‐APC, anti‐CD86‐FITC, anti‐CD40‐PE, anti‐CD3‐APC, anti‐CD4‐FITC, anti‐CD8‐PerCP‐Cy5.5, anti‐FOXP‐3‐PE, anti‐F4/80‐APC, anti‐CD49b‐APC‐eFluor 780, and anti‐CD8‐PerCP‐Cy5.5 were purchased from Invitrogen.

### 4.2 Characterization

The morphology of the samples was characterized by using a field‐emission scanning electron microscope (FE‐SEM, S‐4800, Hitachi) equipped with an energy‐dispersive X‐ray spectrometer. Transmission electron microscopy (TEM) images were obtained on a FEI Tecnai G2 S‐Twin with a field emission gun operating at 200 kV. Fourier transform infrared spectra were measured by IRTracer‐100. High‐Performance Liquid Chromatography (HPLC) was measured by Agilent 1260. Inductively Coupled Plasma (ICP) was taken on an iCAP 6300 of Thermo scientific. ‐DLS and Zeta potential experiments were measured by the Malvern Zeta Sizer‐Nano ZS90 instrument. MTT experiments were carried out using a microplate reader (Thermo, Multiskan MK3). mRNA detections were carried out using RT‐qPCR (Bio‐Rad, CFX96 Touch).

### 4.3 Synthesis of ZIF‐8

12.5 mg of 8‐arm PEG‐OH‐40K was dissolved in 100 µL deionized water under continuous stirring. Sequentially, 250 µL of 2‐methylimidazole aqueous solution (26.28 mg/mL), 50 µL of NaOH aqueous solution (0.2 m), and 450 µL of Zn(NO_3_)_2_ aqueous solution (8.4 mg/mL) were added. The mixture was stirred at room temperature for 3 h. The precipitate was collected by centrifugation (12 000 rpm, 15 min), washed three times with deionized water, and dispersed into 1 mL of deionized water for storage at 4°C.

### 4.4 Synthesis of DZ

12.5 mg of 8‐arm PEG‐OH‐40K was dissolved in 100 µL deionized water under stirring. Subsequently, 250 µL of 2‐methylimidazole aqueous solution (26.28 mg/mL), 50 µL of NaOH aqueous solution (0.2 m), 37.5 µL of 6‐thio‐dG (40 mg/mL), and 450 µL of Zn(NO_3_)_2_ aqueous solution (8.4 mg/mL) were added. The reaction proceeded at room temperature for 3 h. DZ was collected by centrifugation (12 000 rpm, 15 min), washed three times with deionized water, and stored in 1 mL of deionized water at 4°C.

### 4.5 Synthesis of DZT

300 µg of TIM‐3 antibodies was dispersed in 200 µL of 10 mm Tris buffer. Under continuous stirring at 400 rpm, 3 mg of DZ NPs was added dropwise. The mixture was stirred at room temperature for 3 h. The precipitate was collected by centrifugation (12 000 rpm, 15 min), washed three times with deionized water, and stored in 1 mL deionized water at 4°C.

### 4.6 Synthesis of IgG‐Cy5 Modified DZ

10 mg of γ‐IgG was dissolved in 3 mL of NaHCO_3_ aqueous solution (0.1 m), followed by adding 1 mg of Cy5‐NHS. The mixture was stirred at room temperature for 3 h. Cy5‐labeled IgG was purified using a dextran gel chromatography column with Tris elution.1 mg of IgG‐Cy5 was dispersed in 200 µL of 10 mm Tris buffer. Under stirring, 3 mg of DZ NPs was added dropwise. After 3 h of stirring, the product was collected by centrifugation (12 000 rpm, 15 min), washed three times with deionized water, and stored in 1 mL deionized water at 4°C.

### 4.7 In Vitro Release of DZT

DZT was dispersed in Tris‐HCl buffer (pH 7.4 or 6.5) and incubated at 37°C with shaking. Samples were collected at 0, 0.25, 0.5, 1, 2, and 4 h. 6‐thio‐dG release was quantified by UV–vis, and Cy5 fluorescence was measured using a microplate reader.

### 4.8 Stability Evaluation

DZT NPs were dispersed in water, saline, or 10% FBS‐containing DMEM and stored at 4°C. Particle size was measured daily for 7 days using a DLS instrument.

### 4.9 Cell Cultures

MC38, B16‐F10, and 293T cells were purchased from China Center for Type Culture Collection and maintained in DMEM medium with 10% FBS and antibiotics (penicillin 100 U mL^−1^ and streptomycin 100 mg mL^−1^). All cells were cultured at 37°C with 5% CO_2_.

### 4.10 Cell Viability

For cytotoxicity assessment using the CCK‐8 assay, MC38 cells were seeded at 5 × 10^3^ cells per well in 96‐well plates and cultured for 12 h before treatment with PBS, ZIF‐8, 6‐thio‐dG, or DZ for 48 h. CCK‐8 reagent was then added, and viability was calculated by measuring optical density at 450 nm. The same protocol was followed for BMDCs and 293T cells.

### 4.11 Cellular Uptake Assay by CLSM

MC38 cells (4 × 10^5^ cells/well) were seeded in a 6‐well plate and incubated at 37°C with 5% CO_2_ for 24 h. After washing with PBS, the cells were incubated with 30 µL Cy5‐DZ nanoparticles in serum‐free DMEM for 3 h. Then, complete DMEM was added, and cells were incubated for an additional 12 h. After removing the medium, cells were washed 3 times with cold PBS. Hoechst dye was added to stain the nuclei for 10 min, followed by washing with cold PBS. Fluorescence imaging was performed using a fluorescence microscope to observe nanoparticle uptake.

MC38 cells (4×10^5^ cells/well) were seeded and incubated as described above for 24 h. After washing with PBS, cells were incubated with 30 µL Cy5‐DZ nanoparticles in complete DMEM for 6, 12, 24, or 48 h. After incubation, cells were washed 3 times with cold PBS. Then, cells were digested with trypsin and collected, followed by centrifugation at 450 g for 5 min. The supernatant was removed, and cells were washed again with cold PBS. Fluorescence intensity was measured by flow cytometry at different time points.

### 4.12 Apoptosis Assay

MC38 cells (5 × 10^4^ cells/well) were seeded in a 24‐well plate and incubated for 12 h. The culture media were replaced with 1.0 mL of fresh DMEM medium with PBS, ZIF‐8, 6‐thio‐dG, and DZ (C_6‐thio‐dG_ = 800 nm), and cells were incubated for 48 h. After trypsin digestion and centrifugation (500 g, 5 min), cells were washed and resuspended at 1×10^6^ cells/mL. 100 µL of the suspension was mixed with 5 µL Annexin V‐FITC and 5 µL PI, incubated for 15 min, and apoptosis was analyzed by flow cytometry.

### 4.13 Live/Dead Cell Staining

MC38 cells (10×10^4^ cells/well) were seeded in a 24‐well plate and incubated for 12 h. The culture media were replaced with 1.0 mL of fresh DMEM medium with PBS, ZIF‐8, 6‐thio‐dG, and DZ (C_6‐thio‐dG_ = 800 nm), and cells were incubated for 48 h. After washing, cells were stained with 250 µL Calcein AM/PI for 30 min at 37°C in the dark.

### 4.14 Extracellular ATP Level Detection

MC38 cells (10×10^4^ cells/well) were seeded in a 24‐well plate and incubated for 12 h. The culture media were replaced with 1.0 mL of fresh DMEM medium with PBS, ZIF‐8, 6‐thio‐dG, and DZ (C_6‐thio‐dG_ = 800 nm), and cells were incubated for 48 h. After incubation, the supernatant was collected for further analysis. 20 µL of supernatant and 100 µL ATP reagent were mixed in a 96‐well plate, and chemiluminescence was measured using a microplate reader.

### 4.15 Extracellular HMGB1 Level Detection

MC38 cells (10×10^4^ cells/well) were seeded in a 24‐well plate and incubated for 12 h. The culture media were replaced with 1.0 mL of fresh DMEM medium with PBS, ZIF‐8, 6‐thio‐dG and DZ (C_6‐thio‐dG_ = 800 nm), and cells were incubated for 48 h. After incubation, the supernatant was collected for further analysis. 50 µL of supernatant and 100 µL detection antibody were incubated at 4°C for 18 h. After adding 100 µL TMB substrate and incubating for 30 min, the reaction was stopped, and absorbance at 450 nm was measured using a microplate reader. HMGB1 concentration was calculated from a standard curve.

### 4.16 Detection of DNA Damage by CLSM

MC38 cells (10 × 10^4^ cells/well) were seeded in a 24‐well plate and incubated for 12 h. The culture media were replaced with 1.0 mL of fresh DMEM medium with PBS, ZIF‐8, 6‐thio‐dG, and DZ (C_6‐thio‐dG_ = 800 nm). After 48 h of incubation, the treatment medium was removed, and the cells were washed with PBS. Subsequently, the cells were fixed with fixative for 10 min and blocked with blocking solution for 20 min. They were then incubated with a γ‐H2AX primary antibody for 1 h, followed by a corresponding secondary antibody for another hour. Nuclei were stained with DAPI for 5 min. Finally, the cells were visualized using a fluorescence microscope.

### 4.17 Isolation and Culture of BMDCs

According to the method provided in the literature, BMDCs were extracted from the femurs of C57BL/6J mice. The BMDCs were then cultured in RPMI 1640 supplemented with 20 ng/mL GM‐CSF and 10 ng/mL IL‐4 for 7 days after lysis of erythrocytes. The media were replaced with fresh DMEM medium twice, with cytokines added each time. On the seventh day, the BMDCs were collected for further study.

### 4.18 Detection of dsDNA Uptake by BMDCs

MC38 cells (10×10^4^ cells/well) were seeded in a 24‐well plate and incubated for 12 h. Then the DNA fragments were subsequently labeled by co‐incubation with Picogreen‐containing medium for 12 h. The culture media were replaced with 1.0 mL of fresh DMEM medium with PBS, ZIF‐8, α‐TIM‐3, 6‐thio‐dG, DZ, and DZT (C_6‐thio‐dG_ = 800 nm), and cells were incubated for 48 h. BMDCs were seeded at a density of 5×10^6^ cells per well in a 6‐well plate. The supernatant containing MC38 cell fragments, treated with different drugs, was co‐incubated with BMDCs for 24 h. After incubation, the BMDCs were collected, and the uptake of dsDNA was analyzed by flow cytometry.

### 4.19 Western Blot Analysis

MC38 cells (2 × 10^6^ cells/well) were seeded in a 6‐well plate and incubated at 37°C with 5% CO_2_ for 24 h. The culture media were replaced with 1.0 mL of fresh DMEM medium with PBS, ZIF‐8, α‐TIM‐3, 6‐thio‐dG, DZ, and DZT (C_6‐thio‐dG_ = 800 nm), and cells were incubated for 48 h. BMDCs were seeded at a density of 5×10^6^ cells per well in a 6‐well plate. The supernatant containing MC38 cell fragments, treated with different drugs, was co‐incubated with BMDCs for 24 h. After incubation, BMDCs were collected and lysed with RIPA buffer. The cells were then sonicated at 4°C, followed by centrifugation at 4°C (12 000 rpm, 10 min). The supernatant (protein extract) was collected, mixed with sample buffer, and boiled at 100°C for 10 min. The samples can be stored at −80°C. Proteins were separated by SDS‐PAGE and transferred to a PVDF membrane. After blocking with blocking buffer for 15 min, primary antibodies (p‐TBK1, p‐p65, p‐STING, p‐IRF3, and β‐actin) diluted in antibody dilution buffer were incubated overnight at 4°C and then with HRP‐conjugated secondary antibodies for 1 h at room temperature. After washing with TBST, bands were detected using ECL chemiluminescence.

### 4.20 The Maturation of BMDCs

MC38 cells (5 × 10^4^ cells/well) were seeded in a 24‐well plate and incubated at 37°C with 5% CO_2_ for 12 h. The culture media were replaced with 1.0 mL of fresh DMEM medium with PBS, ZIF‐8, α‐TIM‐3, 6‐thio‐dG, DZ, and DZT (C_6‐thio‐dG_ = 800 nm), and cells were incubated for 48 h. BMDCs were seeded at a density of 5×10^5^ cells per well in a 24‐well plate. The supernatant containing MC38 cell fragments, treated with different drugs, was co‐incubated with BMDCs for 24 h. Finally, cells and supernatants were collected. Cells were stained with anti‐CD45‐Alex Fluor 700, anti‐CD11c‐PerCP‐Cy5.5, anti‐CD80‐APC, anti‐CD86‐FITC, and anti‐CD40‐PE antibodies for fluorescence labeling and analyzed by flow cytometry.

### 4.21 In Vitro IFN‐β Analysis

The collected cell supernatant was tested using an IFN‐β ELISA kit, with blank, standard, and sample wells set up. 100 µL of the sample solution was added to the IFN‐β antibody‐coated microplate and incubated at 37°C for 90 min. Biotinylated antibody, horseradish peroxidase (HRP), and TMB substrate were added sequentially and incubated for the appropriate time. Finally, a stop solution was added, and absorbance at 450 nm was measured using a microplate reader. A standard curve was plotted to calculate the IFN‐β concentration in the samples.

### 4.22 RNA Sequencing of BMDCs

MC38 cells (2 × 10^6^ cells/well) were seeded in a 6‐well plate and incubated at 37°C with 5% CO_2_ for 24 h. BMDCs were seeded at a density of 5 × 10^6^ cells per well in a 6‐well plate. The supernatant containing MC38 cell fragments, treated with different drugs, was co‐incubated with BMDCs for 24 h. BMDCs were collected, rapidly frozen in liquid nitrogen, and stored at −80°C. RNA sequencing was performed based on the Illumina sequencing platform.

### 4.23 Hemolytic Assay

Whole blood (0.5 mL) was collected from healthy mice via retro‐orbital bleeding and centrifuged at 3000 rpm for 15 min. The supernatant was discarded, and erythrocytes were washed twice with PBS until the supernatant became clear. The pelleted red blood cells (RBCs) were then diluted with 10 mL of physiological saline (0.9% NaCl) to prepare a 2% (v/v) RBC suspension (0.2 mL packed RBCs in 10 mL saline). Aliquots (1 mL) of the RBC suspension were mixed with 0.1% Triton X‐100 (positive control), PBS (negative control), and different groups, then incubated at 37°C for 1 h. After incubation, samples were centrifuged (3000 rpm, 15 min), and the supernatant was photographed for visual documentation. Hemoglobin release was quantified by measuring the absorbance of the supernatant at 540 nm using a microplate reader. The hemolysis ratio (%) was calculated as: Hemolysis ratio (%) = (OD_x_ ‐OD_negative_) / (OD_positive_—OD_negative_) ×100%.

### 4.24 Animals

C57BL/6 aged 6–8 weeks (∼18 g) were purchased from the GemPharmatech Co., Ltd. All mice were treated in accordance with the approved protocol of the Institutional Animal Care and Use Committee of Peking Union Medical College (Ethics Number: IMM‐S‐25‐0445). All mice were kept under a tightly controlled temperature (22°C), humidity (40%–50%), and light/dark (12/12 h) cycle conditions with unrestricted access to water and food.

### 4.25 Biosafety Evaluation In Vivo

Healthy C57bl/6J mice were used to evaluate the in vivo biosafety. DZT (10 mg/kg) was administered intravenously every 3 days, and the mice's body weight was recorded. On days 7 and 14, serum was collected for liver and kidney function tests (n = 3). After 14 days, the mice were sacrificed, and their major organs (heart, liver, spleen, lungs, and kidneys) were dissected for H&E staining.

### 4.26 Biodistribution of IgG‐Cy5@DZ In Vivo

IgG‐Cy5@DZ, synthesized by the aforementioned method, was used to evaluate the systemic distribution of the DZT and its targeting effect at the tumor site. A subcutaneous CT‐26 tumor model in mice was established, and when the tumor volume reached approximately 200 mm^3^, each mouse was injected with IgG‐Cy5@DZ. At different time points after administration (0, 8, 12, and 24 h), the mice were anesthetized with isoflurane, and fluorescence imaging was performed using a small animal in vivo imaging system to monitor the Cy5 signal in real‐time, assessing the distribution of the nanodrug at the tumor site. After 24 h, the mice were anesthetized, underwent cardiac perfusion, and were then humanely euthanized. The heart, liver, spleen, lungs, kidneys, and tumor tissues were dissected, and fluorescence signals from *ex vivo* organs were detected using the in vivo imaging system.

### 4.27 In Vivo Antitumor Effect and Immune Mechanism

To establish the subcutaneous MC38 tumor model in mice, 1.3 × 10^6^ cells were injected into C57BL/6 mice. When the tumor volume reached 50 mm^3^, the mice were randomly divided into 6 groups, with 10 mice per group. The dosing regimen was as follows: PBS, ZIF‐8, α‐TIM‐3, 6‐thio‐dG, DZ, and DZT (6‐thio‐dG: 430 µg/kg, α‐TIM‐3: 1.5 mg/kg, ZIF‐8: 6.65 mg/kg) were administered via tail vein injection on days 0, 3, 6, and 9. Tumor volume and body weight of the mice were measured and recorded regularly every other day. On day 14 after treatment, half of the mice from each group were humanely euthanized, and the spleen, tumor‐draining lymph nodes, and tumor were collected for further experimental studies. Additionally, heart, liver, spleen, lung, kidney, and tumor tissues were collected for H&E staining. The remaining half of the mice were continued to be monitored for survival. Cells were stained using anti‐CD45‐Alex Fluor 700, anti‐CD3‐APC, anti‐CD4‐FITC, anti‐CD8‐PerCP‐Cy5.5, anti‐F4/80‐APC, anti‐CD80‐APC, anti‐CD206‐PE, anti‐CD49b‐APC‐eFluor 780, and anti‐CD107a‐PE to analyze the levels of T cells, TAMs, and NK cells within the tumor. Subsequently, tumor supernatant was used to measure the cytokine secretion levels of IFN‐β, IFN‐γ, DZMB, and TNF‐α. Cells were stained with anti‐CD11c‐PE‐Cy5.5, anti‐CD80‐APC, anti‐CD86‐FITC, anti‐CD3‐APC, anti‐CD4‐FITC, and anti‐CD8‐PerCP‐Cy5.5 to analyze the levels of DCs and T cells in the lymph nodes. Cells were stained using anti‐CD3‐APC, anti‐CD4‐FITC, and anti‐CD8‐PerCP‐Cy5.5 to analyze the levels of T cells in the spleen. Finally, immunofluorescence staining was performed on tumor tissue using CD8 antibody and TUNEL staining to analyze the presence of CD8^+^ T cells and apoptotic cells.

### 4.28 In Vivo Antitumor Effect and Immune Mechanism

To establish the subcutaneous B16F10 tumor model in mice, 9 × 10^5^ cells were injected into C57BL/6 mice. The mice were randomly divided into 6 groups, with 5 mice per group. The dosing regimen was as follows: PBS, ZIF‐8, αTIM‐3, 6‐thio‐dG, DZ, and DZT (6‐thio‐dG: 430 µg/kg, αTIM‐3: 1.5 mg/kg, ZIF‐8: 6.65 mg/kg) were administered via tail vein injection on days 0, 3, 6, and 9. Tumor volume and body weight of the mice were measured and recorded regularly every other day. On day 14 after treatment, the mice were humanely euthanized, and the spleen, tumor‐draining lymph nodes, and tumor were collected for further experimental studies. Additionally, heart, liver, spleen, lung, kidney, and tumor tissues were collected for H&E staining. Cells were stained using anti‐CD45‐Alex Fluor 700, anti‐CD3‐APC, anti‐CD4‐FITC, anti‐CD8‐PerCP‐Cy5.5, anti‐F4/80‐APC, anti‐CD80‐APC, anti‐CD40‐PE, anti‐CD206‐PE, and anti‐FOXP‐3‐PE to analyze the levels of T cells, DCs, TAMs, and Treg cells within the tumor. Subsequently, tumor supernatant was used to measure the cytokine secretion levels of IFN‐β, IFN‐γ, DZMB, and TNF‐α. Cells were stained with anti‐CD11c‐PE‐Cy5.5, anti‐CD80‐APC, anti‐CD86‐FITC, anti‐CD40‐PE, anti‐CD3‐APC, anti‐CD4‐FITC, and anti‐CD8‐PerCP‐Cy5.5 to analyze the levels of DCs and T cells in the lymph nodes. Cells were stained using anti‐CD3‐APC, anti‐CD4‐FITC, anti‐CD8‐PerCP‐Cy5.5, anti‐CD49b‐APC‐eFluor 780, and anti‐CD107a‐PE to analyze the levels of T cells and NK cells in the spleen. Finally, immunofluorescence staining was performed on tumor tissue using CD8 antibody and TUNEL staining to analyze the presence of CD8^+^ T cells and apoptotic cells.

### 4.29 Organoid Culture and Co‐Incubation with BMDCs

The frozen breast cancer organoids were thawed at 37°C and centrifuged at 1500 rpm for 5 min. The cell aggregates were then resuspended in 50% cold Matrigel, and 50 µL droplets were seeded onto pre‐warmed 96‐well plates. The droplets were allowed to solidify for 30 min in a 37°C incubator with 5% CO_2_. After solidification, 200 µL of organoid culture medium was added to each well, and the medium was refreshed every 2–3 days.

When the number of cell aggregates in each well reaches approximately 20, the culture media were replaced with 1.0 mL of fresh organoid culture medium with PBS, ZIF‐8, α‐TIM‐3, 6‐thio‐dG, DZ, and DZT (C_6‐thio‐dG_ = 50 µg/mL), and cells were incubated for 48 h. BMDCs were seeded at a density of 1 × 10^6^ cells per well in a 24‐well plate. The supernatant containing MC38 cell fragments, treated with different drugs, was co‐incubated with BMDCs for 24 h. Finally, cells and supernatants were collected. Cells were stained with anti‐CD45‐Alex Fluor 700, anti‐CD11c‐PerCP‐Cy5.5, anti‐CD80‐APC, anti‐CD86‐FITC, and anti‐CD40‐PE antibodies for fluorescence labeling and analyzed by flow cytometry. The collected cell supernatant was tested using an IFN‐β ELISA kit, with blank, standard, and sample wells set up. 100 µL of the sample solution was added to the IFN‐β antibody‐coated microplate and incubated at 37°C for 90 min. Biotinylated antibody, horseradish peroxidase (HRP), and TMB substrate were added sequentially and incubated for the appropriate time. Finally, a stop solution was added, and absorbance at 450 nm was measured using a microplate reader.

### 4.30 Statistical Analysis

All data were presented as mean ± SD, and analyzed using GraphPad Prism 8. The significance of the differences among groups was calculated by one‐way analysis of variance (ANOVA), followed by Tukey's multiple comparisons test for post hoc analysis. Statistical significance was denoted as ^*^ *p* < 0.05, ^**^ *p* < 0.01, ^***^ *p* < 0.001, ^****^ *p* < 0.0001.

## Conflicts of Interest

The authors declare no conflicts of interest.

## Supporting information




**Supporting File**: advs73827‐sup‐0001‐SuppMat.docx.

## Data Availability

The data that support the findings of this study are available from the corresponding author upon reasonable request.
