# Supplementary material for: A Programmable Nanoreactor Orchestrates Cascade of DNA Sensing to Amplify cGAS‐STING Activation for Cancer Immunotherapy
Source: Adv Sci (Weinh). 2026 Jan 20;13(17):e18356. doi: 10.1002/advs.202518356 (PMC13042556; doi:10.1002/advs.202518356)
Supplement: Supplementary file 1 — Supporting File: advs73827‐sup‐0001‐SuppMat.docx. [file ADVS-13-e18356-s001.docx]

Supporting Information

**A Programmable Nanoreactor Orchestrates Cascade of DNA Sensing to Amplify cGAS-STING Activation for Cancer Immunotherapy**

*Shuang Liang, Yiwei Tian, Feiyu Zhao, Yue Han, Kongshuo Ma, Linna Hai, Kaiqing Yun, Yueyang Zhao, Siqi Zhang, Ziyi Zhang, Yuxuan Peng, Kuan Hu, Jing Zhong*, Bai Xiang*, Zhaohui Wang**

S. Liang, K, Ma, L. Hai, K. Yun, Dr. S. Zhang, Z. Zhang, Y. Han, Y. Peng, K. Hu, Z. Wang

State Key Laboratory of Bioactive Substance and Function of Natural Medicines, Institute of Materia Medica, Chinese Academy of Medical Sciences & Peking Union Medical College, Beijing 100050, P. R. China

E-mail: [zhaohuiwang@imm.ac.cn](mailto:zhaohuiwang@imm.ac.cn)

S. Liang, K, Ma, L. Hai, K. Yun, Z. Zhang, Y. Han, Y. Peng, Z. Wang

Beijing Key Laboratory of Key Technologies for Natural Drug Delivery and Novel Formulations, Institute of Materia Medica, Chinese Academy of Medical Sciences & Peking Union Medical College, Beijing 100050, China

Y, Tian, Y, Zhao, B. Xiang

Hebei Key Laboratory of Innovative Drug Research and Evaluation, School of Pharmaceutical Sciences, Hebei Medical University, Shijiazhuang 050017, P. R. China

E-mail: [baixiang@hebmu.edu.cn](mailto:baixiang@hebmu.edu.cn)

F. Zhao

Department of Radiation Therapy, Oncology Medical Department, the Fifth Medical Center of Chinese People's Liberation Army General Hospital, No. 8 East Main Street, Fengtai District, Beijing, 100071, P. R. China.

J. Zhong

School of Instrumentation and Opto-Electronic Engineering, Ministry of Education Key Laboratory of Precision Opto-Mechatronics Technology, Beihang University, Beijing 100191, P. R. China

E-mail: zhongjing@buaa.edu.cn


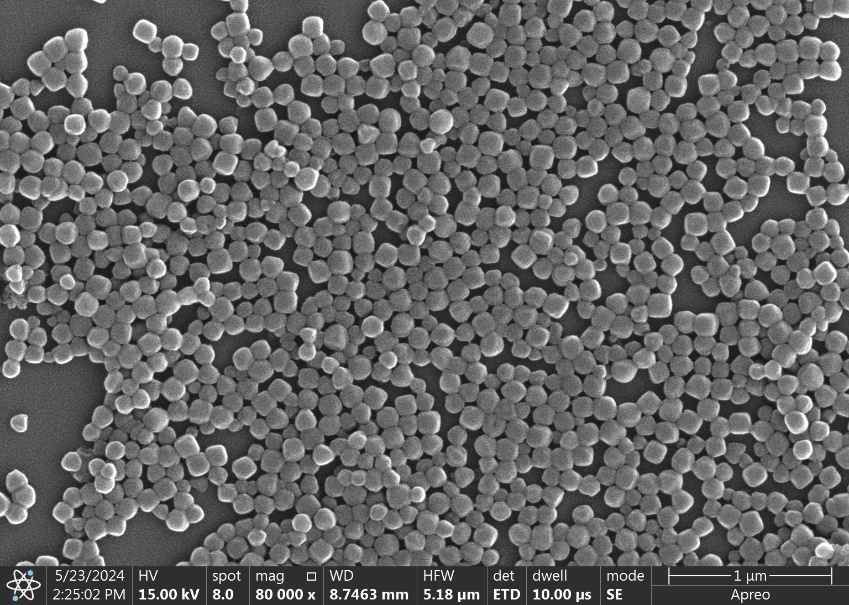


Figure S1. The SEM image of DZ NPs. Scale bar = 500 nm.


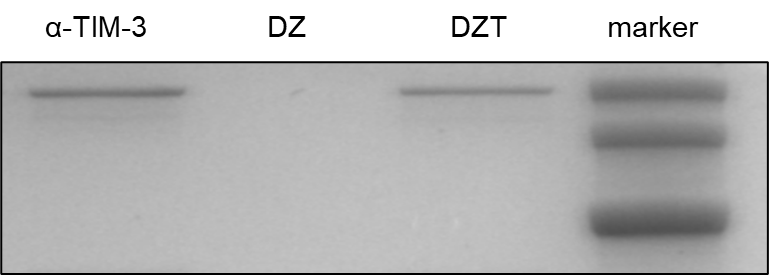


Figure S2. Evaluation of αTIM-3 loading eﬃciency by gel electrophoresis.

Figure S3. X-ray diffraction patterns of DZT.

Figure S4. Hydrodynamic size of DZT dispersed in water, saline, and DMEM during 7 days. Data were expressed as means ± SD (n = 3).

Figure S5. αTIM-3 release profile of DZT cultured with different conditions (pH 7.4 and 6.5). Data were expressed as means ± SD (n = 3).

**
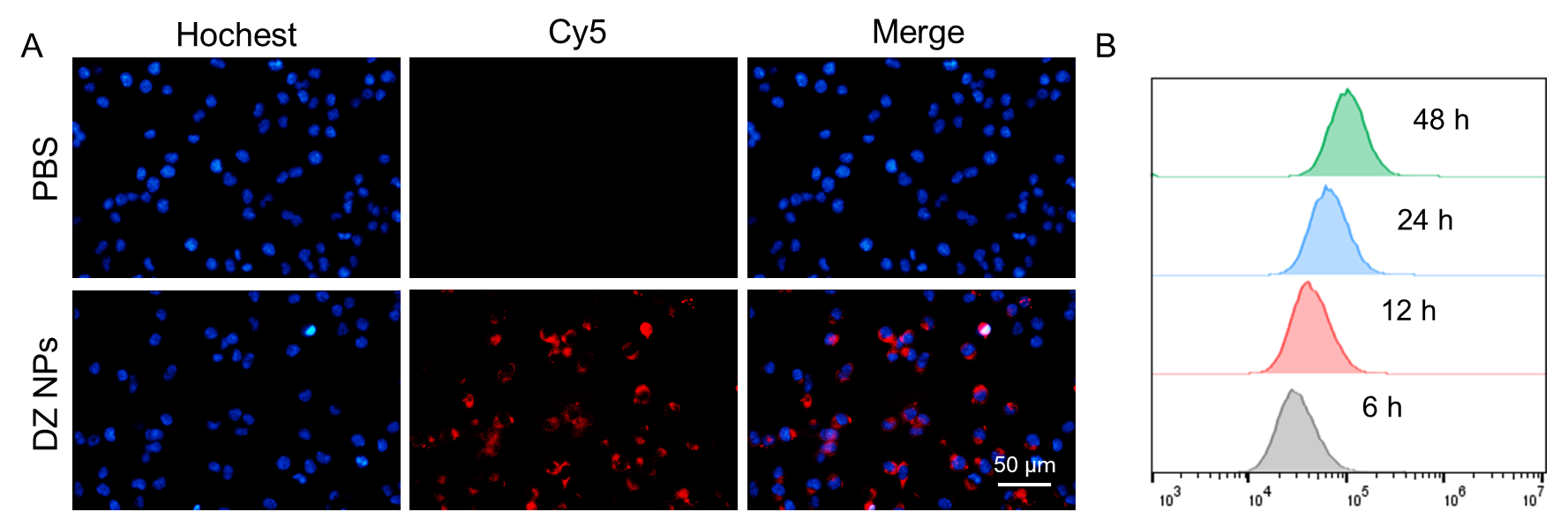
**

Figure S6. The fluorescent imaging (A) and flow cytometry analysis (B) of cellular uptake of DZ into MC38 cells.

Figure S7. Flow cytometry analysis of the uptake mechanism of DZ NPs on MC38 cells. Data are shown as mean ± SD (n = 3). Statistical analysis was measured by one-way ANOVA, * *p* < 0.05, ** *p* < 0.01, and *** *p* < 0.001.

**
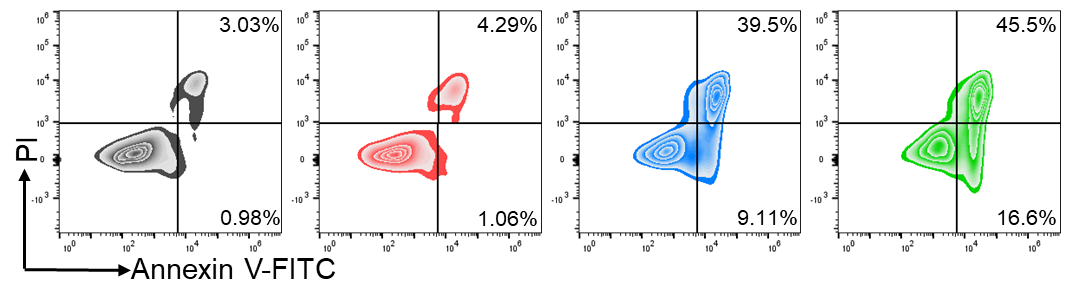
**

Figure S8. Flow cytometry analysis of MC38 cells apoptosis status after treatment with PBS, ZIF-8, 6-thio-dG, and DZ NPs.

**
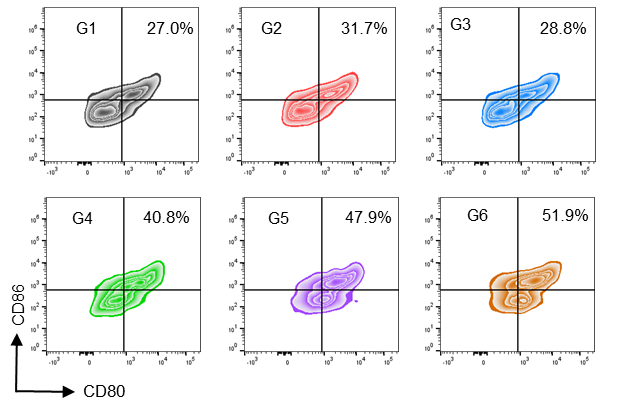
**

Figure S9. Flow cytometry analysis of BMDC maturation after co-incubation with organoids treated with various treatments including G1: PBS, G2: ZIF-8, G3: αTIM-3, G4: 6-thio-dG, G5: DZ NPs, G6: DZT NPs.

**
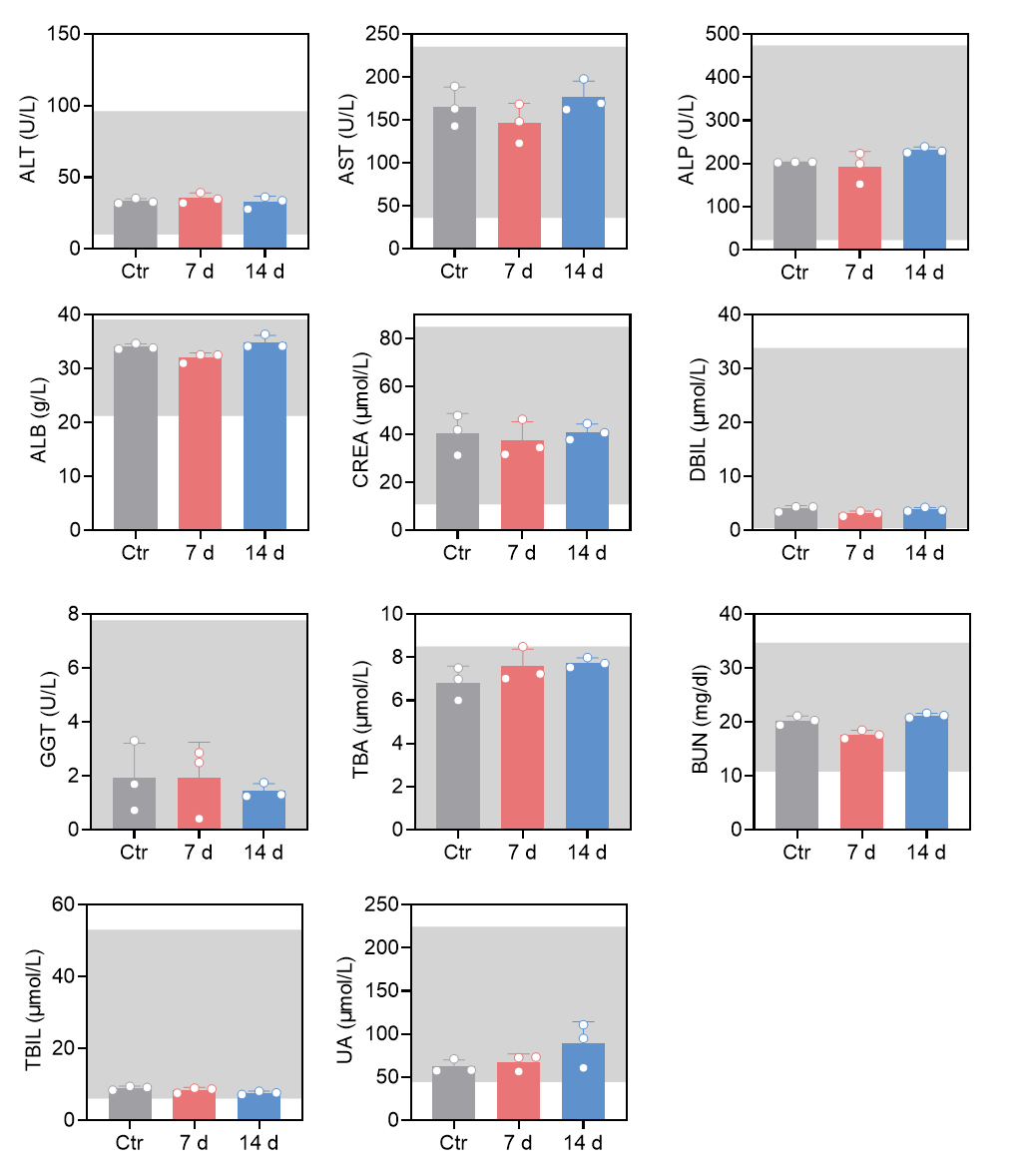
**

Figure S10. Hematological indexes of the mice sacrificed at 0, 7, and 14 days after treatment with DZT NPs. Data are shown as means ± SD (n = 3).

Figure S11. The body weight of C57BL/6 mice after treatment with DZT NPs. Data are shown as means ± SD (n = 3).


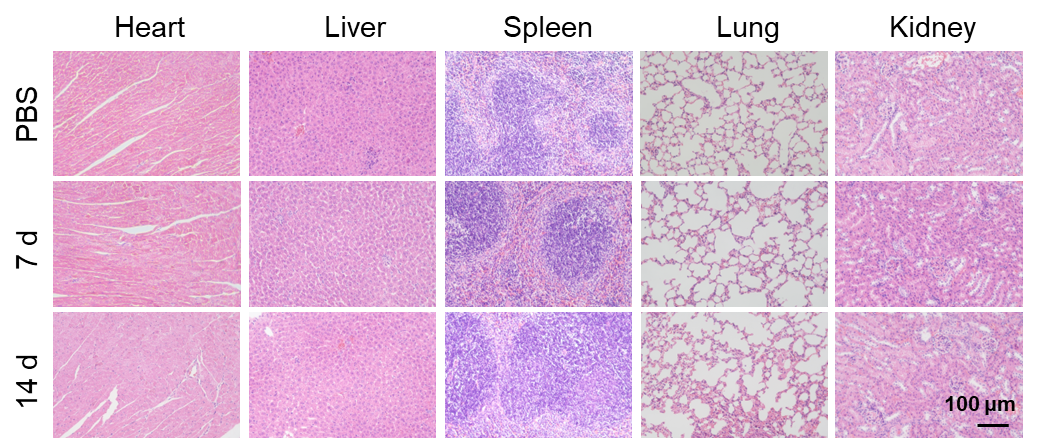


Figure S12. H&E-stained slice images of heart, liver, spleen, and kidney of C57BL/6 mice after treatment with DZT NPs.


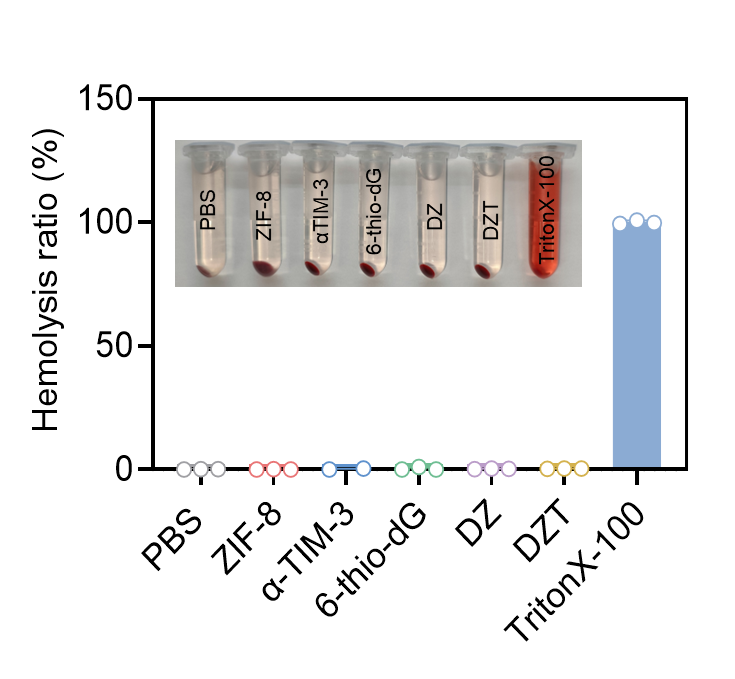


Figure S13. The hemolysis rates of different groups. Data are shown as means ± SD (n = 3).


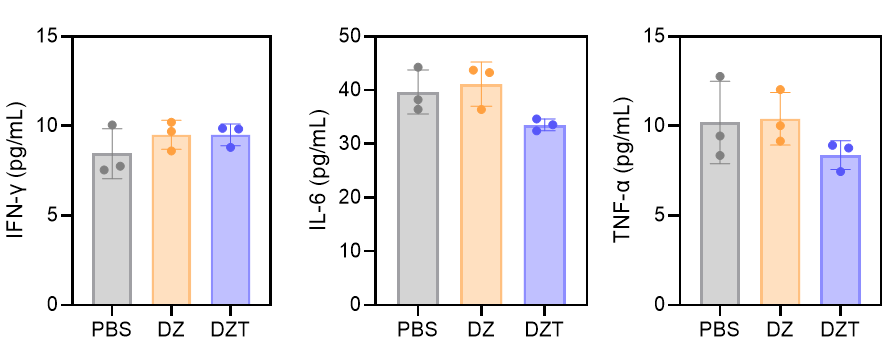


Figure S14. Serum levels of the proinflammatory cytokine IFN-*γ*, IL-6, and TNF-α at 1 h post-injection of DZ and DZT. Data are shown as means ± SD (n = 3).


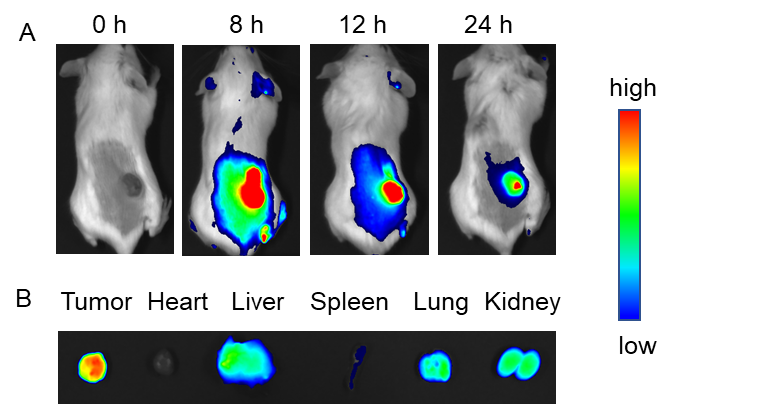


Figure S15. (A) *In vivo* imaging of tumor bearing mice at different time points after intravenous injection of Cy5-labeled DZT NPs. (B) *Ex vivo* imaging of major organs and tumors post intravenous injection of Cy5-labeled DZT NPs for 24 h.


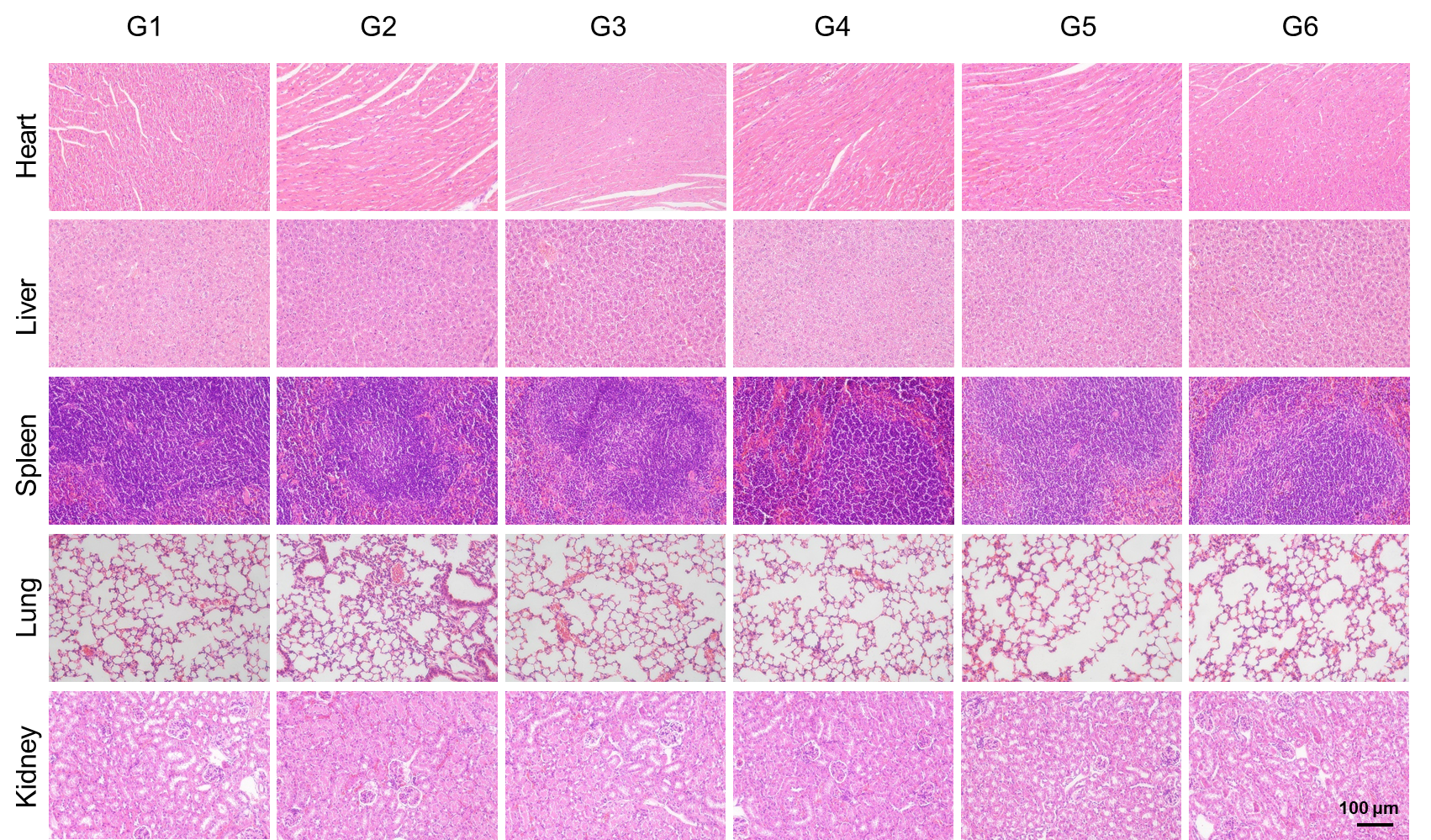


Figure S16. H&E-stained slice images of heart, liver, spleen, and kidney of MC38 tumor-bearing mice after different treatments including G1: PBS, G2: ZIF-8, G3: αTIM-3, G4: 6-thio-dG, G5: DZ NPs, G6: DZT NPs.


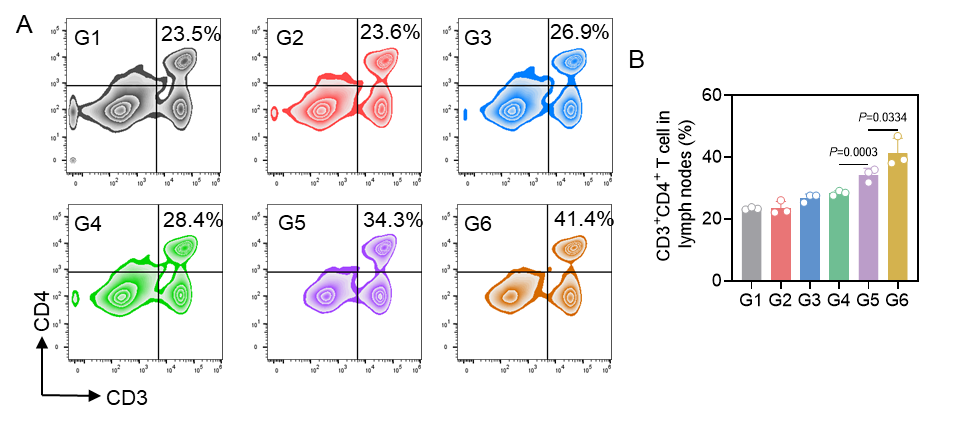


Figure S17. Representative flow cytometric analysis (A) and quantification (B) of CD8^+^ T cells in the dLNs after different treatments including G1: PBS, G2: ZIF-8, G3: αTIM-3, G4: 6-thio-dG, G5: DZ NPs, G6: DZT NPs. Data are shown as mean ± SD (n = 3). Statistical analysis was measured by one-way ANOVA, * *p* < 0.05, ** *p* < 0.01, and *** *p* < 0.001.


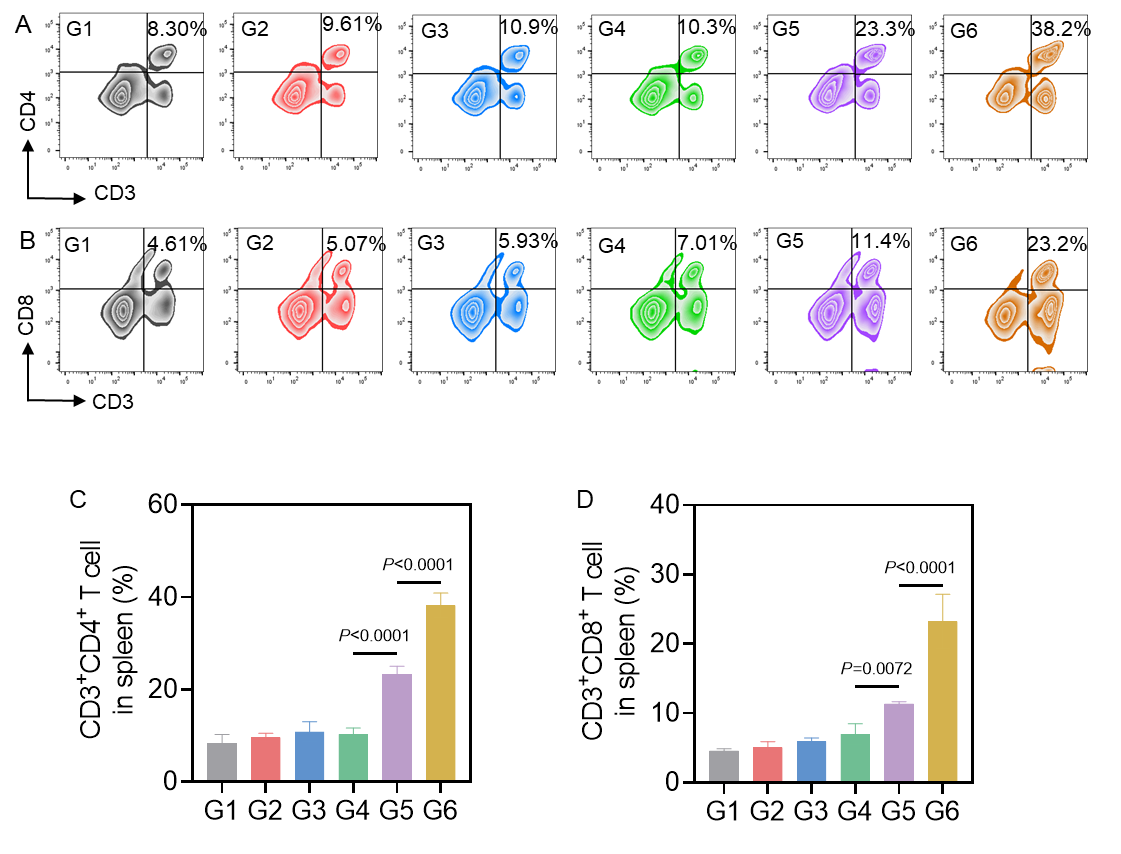


Figure S18. Representative flow cytometric analysis (A, B) and quantification (C, D) of CD4^+^ and CD8^+^ T cells in the spleen after different treatments including G1: PBS, G2: ZIF-8, G3: αTIM-3, G4: 6-thio-dG, G5: DZ NPs, G6: DZT NPs. Data are shown as mean ± SD (n = 3). Statistical analysis was measured by one-way ANOVA, * *p* < 0.05, ** *p* < 0.01, and *** *p* < 0.001.


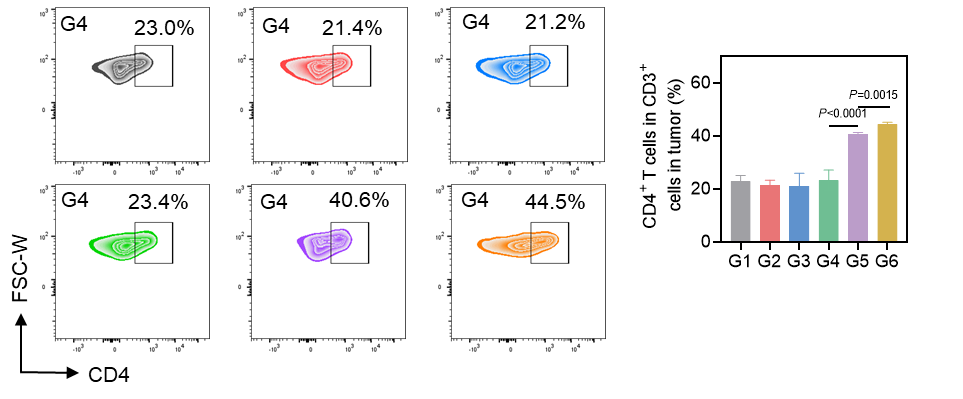


Figure S19. Representative flow cytometric analysis and quantification of CD4^+^ T cells in the tumor after different treatments including G1: PBS, G2: ZIF-8, G3: αTIM-3, G4: 6-thio-dG, G5: DZ NPs, G6: DZT NPs. Data are shown as mean ± SD (n = 3). Statistical analysis was measured by one-way ANOVA, * *p* < 0.05, ** *p* < 0.01, and *** *p* < 0.001.


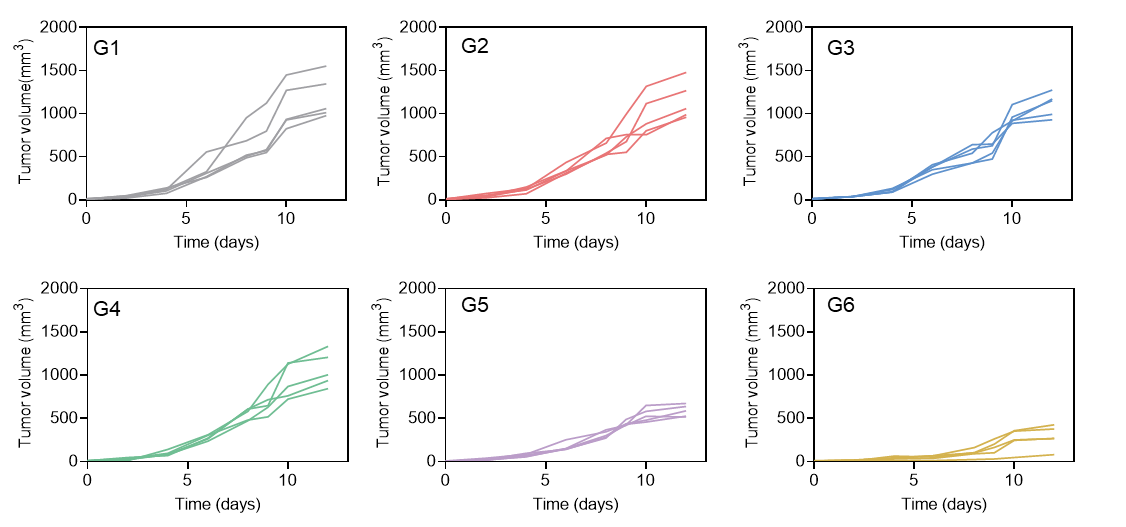


Figure S20. Individual tumor growth curves of B16F10 tumor-bearing mice with different treatments including G1: PBS, G2: ZIF-8, G3: αTIM-3, G4: 6-thio-dG, G5: DZ NPs, G6: DZT NPs.


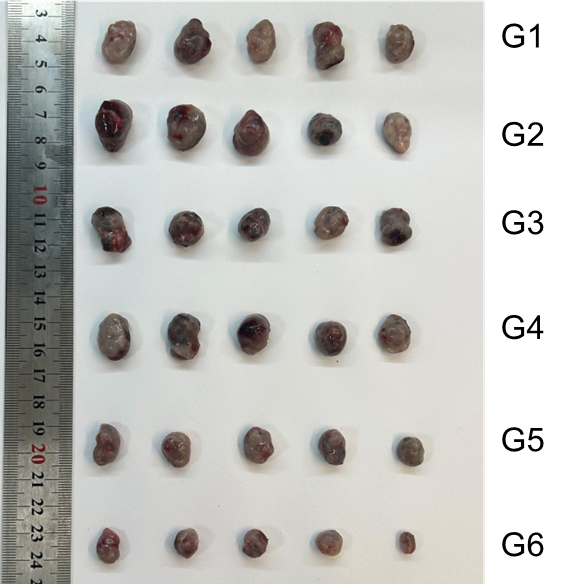


Figure S21. Photograph of dissected tumors in B16F10 tumor-bearing mice after different treatments including G1: PBS, G2: ZIF-8, G3: αTIM-3, G4: 6-thio-dG, G5: DZ NPs, G6: DZT NPs.


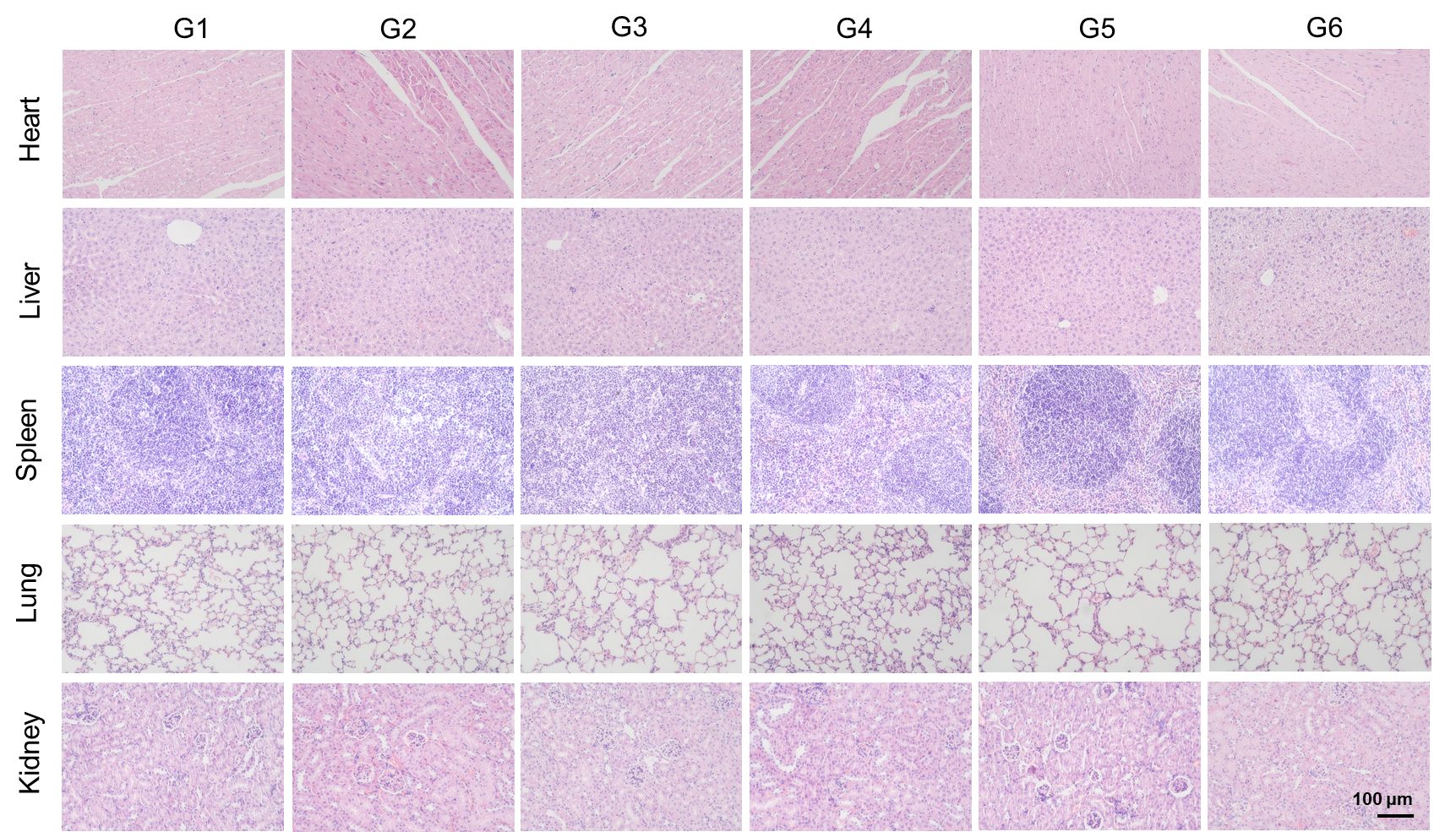


Figure S22. H&E-stained slice images of heart, liver, spleen, lung, and kidney of B16F10 tumor-bearing mice after different treatments including G1: PBS, G2: ZIF-8, G3: αTIM-3, G4: 6-thio-dG, G5: DZ NPs, G6: DZT NPs.

Figure S23. The body weight of B16F10 tumor-bearing mice during the treatments including G1: PBS, G2: ZIF-8, G3: αTIM-3, G4: 6-thio-dG, G5: DZ NPs, G6: DZT NPs. Data are shown as mean ± SD (n = 3).


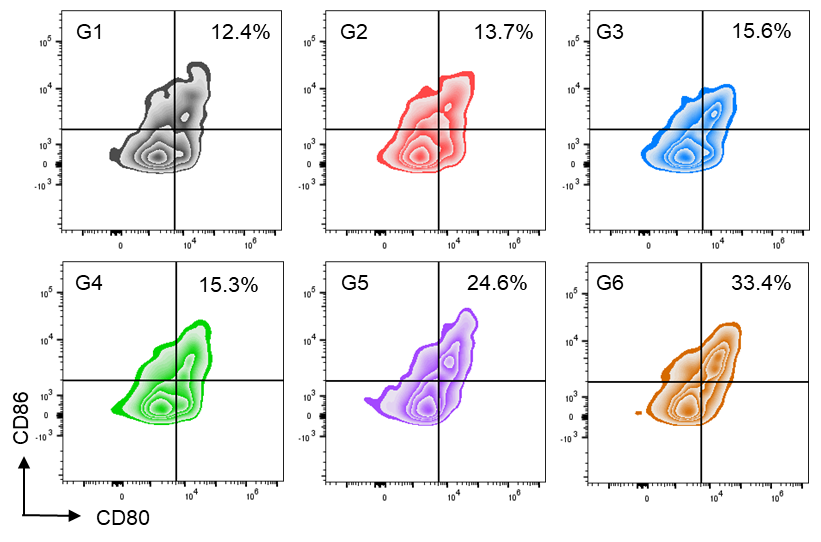


Figure S24. Flow cytometric assay of mature DCs in the dLNs after different treatments including G1: PBS, G2: ZIF-8, G3: αTIM-3, G4: 6-thio-dG, G5: DZ NPs, G6: DZT NPs.


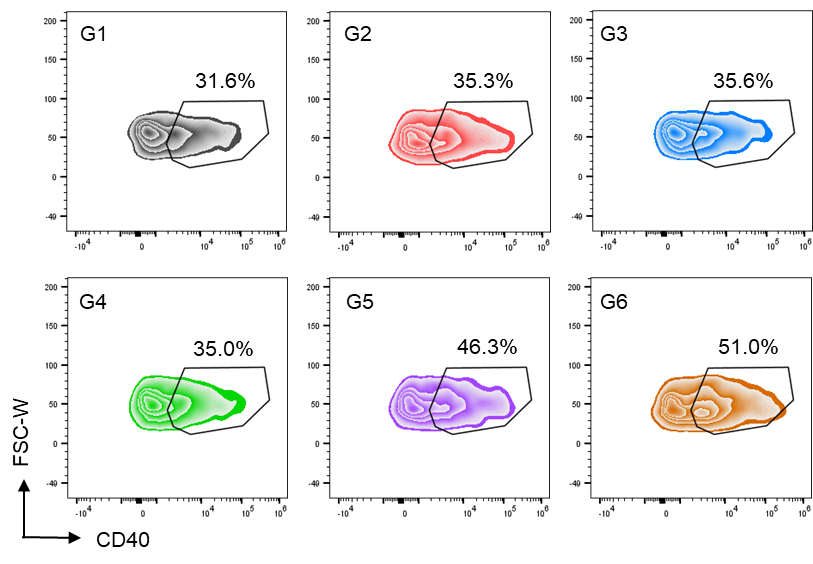


Figure S25. Flow cytometric assay of mature DCs in the dLNs after different treatments including G1: PBS, G2: ZIF-8, G3: αTIM-3, G4: 6-thio-dG, G5: DZ NPs, G6: DZT NPs.


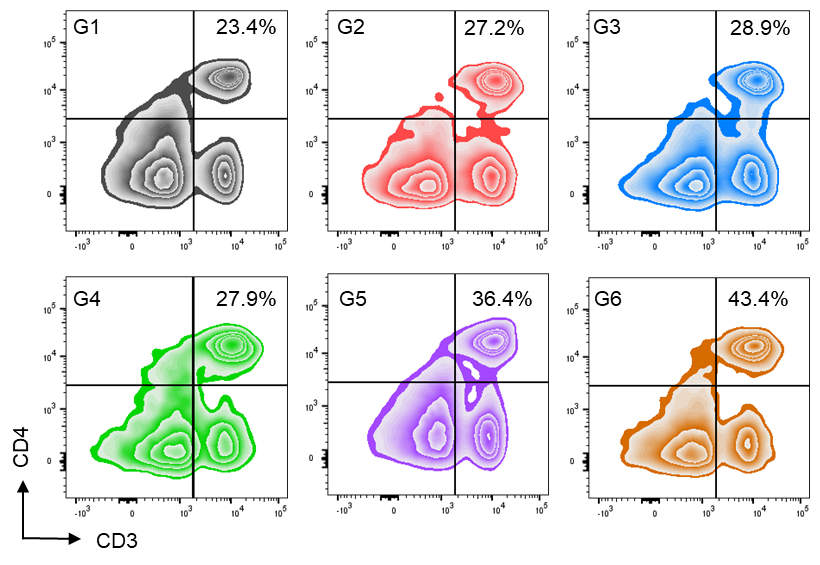


Figure S26. Flow cytometric assay of CD4^+^ T cell in the dLNs after different treatments including G1: PBS, G2: ZIF-8, G3: αTIM-3, G4: 6-thio-dG, G5: DZ NPs, G6: DZT NPs.


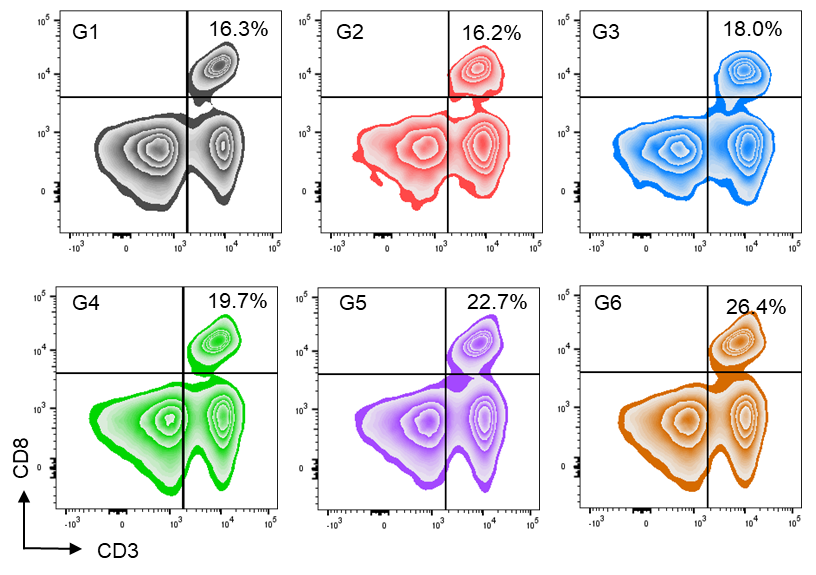


Figure S27. Flow cytometric assay of CD8^+^ T cell in the dLNs after different treatments including G1: PBS, G2: ZIF-8, G3: αTIM-3, G4: 6-thio-dG, G5: DZ NPs, G6: DZT NPs.


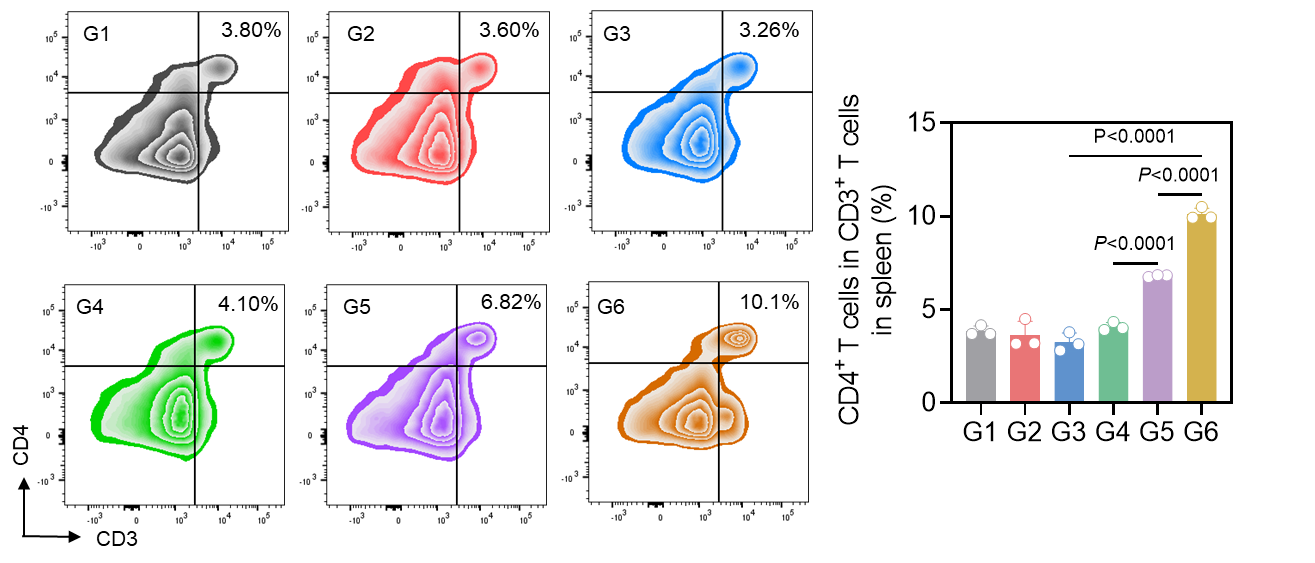


Figure S28. Representative flow cytometric analysis and quantification of CD4^+^ cells in the spleen after different treatments including G1: PBS, G2: ZIF-8, G3: αTIM-3, G4: 6-thio-dG, G5: DZ NPs, G6: DZT NPs. Data are shown as mean ± SD (n = 3). Statistical analysis was measured by one-way ANOVA, * *p* < 0.05, ** *p* < 0.01, and *** *p* < 0.001.


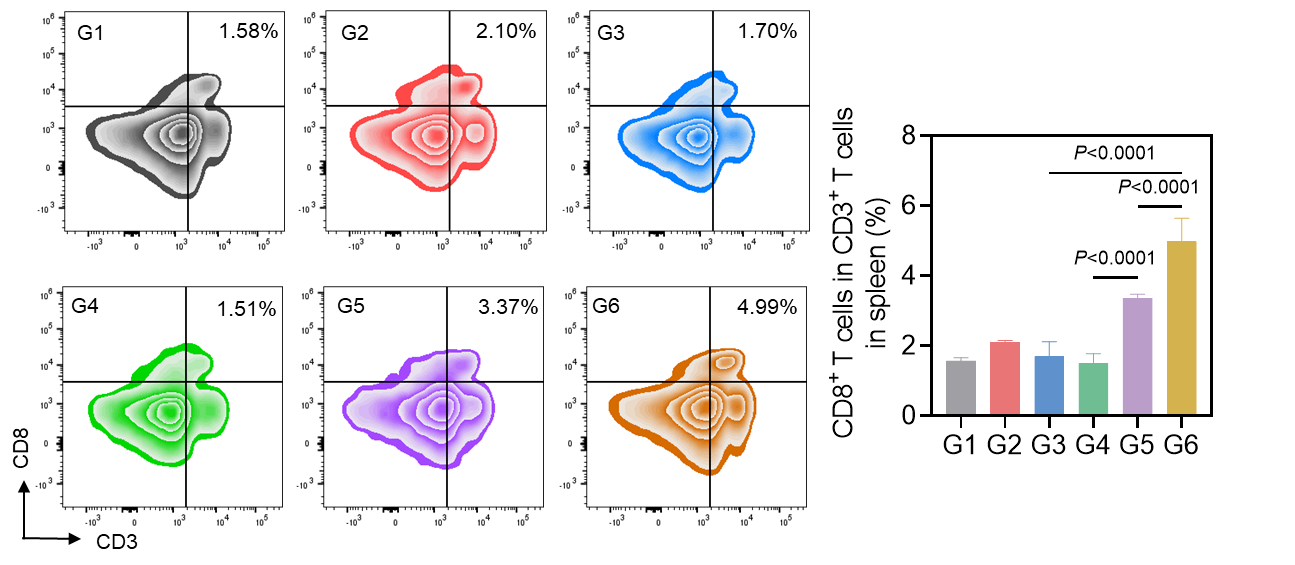


Figure S29. Representative flow cytometric analysis and quantification of CD8^+^ cells in the spleen after different treatments including G1: PBS, G2: ZIF-8, G3: αTIM-3, G4: 6-thio-dG, G5: DZ NPs, G6: DZT NPs. Data are shown as mean ± SD (n = 3). Statistical analysis was measured by one-way ANOVA, * *p* < 0.05, ** *p* < 0.01, and *** *p* < 0.001.


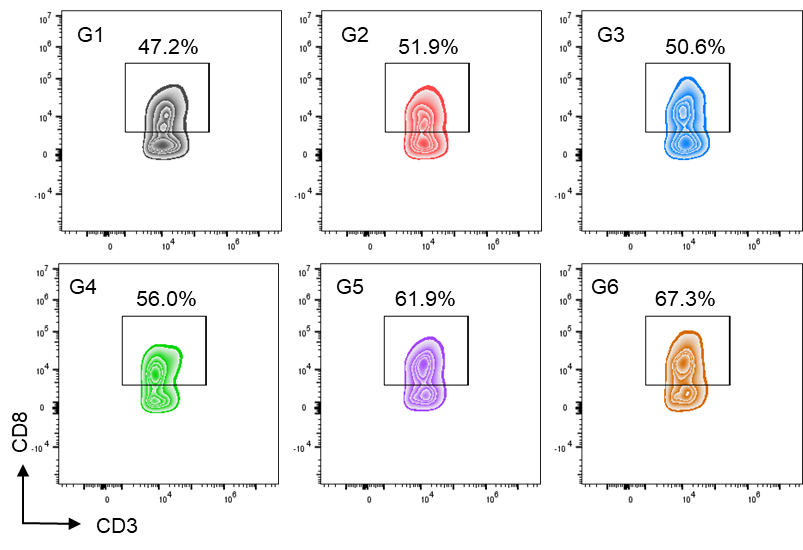


Figure S30. Flow cytometric assay of CD8^+^ T cell in the TME after different treatments including G1: PBS, G2: ZIF-8, G3: αTIM-3, G4: 6-thio-dG, G5: DZ NPs, G6: DZT NPs.


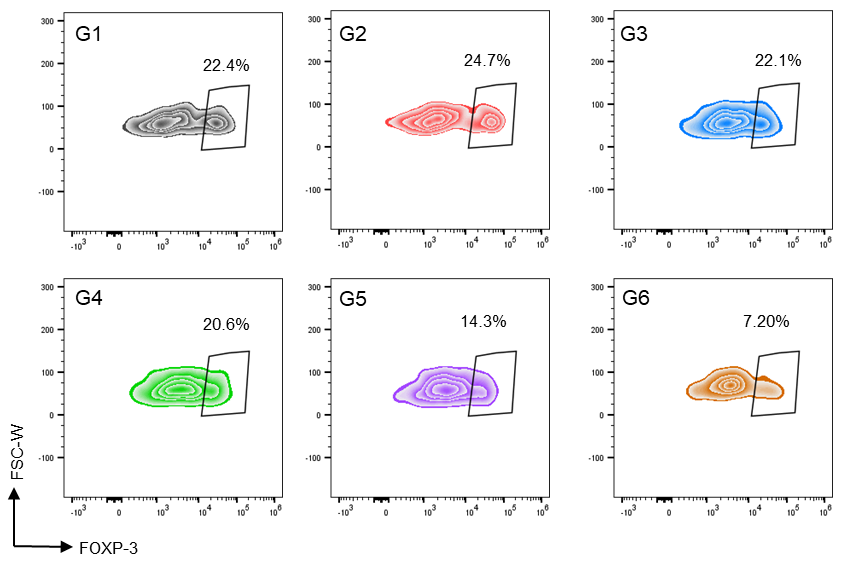


Figure S31. Flow cytometric assay of Tregs in the TME after different treatments including G1: PBS, G2: ZIF-8, G3: αTIM-3, G4: 6-thio-dG, G5: DZ NPs, G6: DZT NPs.
